# Adipocyte-activated oxidative and ER stress pathways promote tumor survival in bone via upregulation of Heme Oxygenase 1 and Survivin

**DOI:** 10.1038/s41598-017-17800-5

**Published:** 2018-01-08

**Authors:** Mackenzie K. Herroon, Erandi Rajagurubandara, Jonathan D. Diedrich, Elisabeth I. Heath, Izabela Podgorski

**Affiliations:** 1 Department of Pharmacology, Wayne State University School of Medicine, Detroit, MI USA; 2 Oncology, Wayne State University School of Medicine, Detroit, MI USA; 3 Karmanos Cancer Institute, Wayne State University School of Medicine, Detroit, MI USA

## Abstract

Metastatic tumor cells engage the local tumor microenvironment and activate specific pro-survival mechanisms to thrive and progress in the harsh bone marrow niche. Here we show that the major contributors to the survival of carcinoma cells that have colonized the bone marrow are the adipocyte-induced oxidative stress and ER stress pathways. We demonstrate that upon exposure to adipocyte-rich environments *in vitro* or *in vivo*, bone-trophic prostate and breast tumor cells upregulate the oxidative stress enzyme, HO-1. We also show that HO-1 levels are significantly increased in human metastatic prostate cancer tissues and that stable HO-1 overexpression in tumor cells promotes growth and invasiveness. Co-incident with the adipocyte-induced expression of HO-1, there is an upregulation of ER chaperone BIP and splicing of XBP1, indicating adipocyte-driven unfolded protein response, a process that we show to be sensitive to antioxidant treatment. Importantly, we also demonstrate that triggering of the oxidative stress and ER stress responses, or HO-1 induction by adipocyte exposure result in the activation of pro-survival pathways, involving survivin. Collectively, our findings reveal a new link between HO-1 and survivin expression in tumor cells, and provide a new insight into potentially targetable survival pathways in bone-metastatic disease.

## Introduction

Bone is a preferred site of metastasis from several solid cancers, particularly tumors of prostate and breast, known for high frequency of skeletal lesions upon recurrence^1,2^. Effective treatment of skeletal disease in metastatic patients is an unmet clinical need, as the mechanisms driving progression and survival in bone are still not well understood. The survival of bone-trophic tumors in harsh metastatic environments such as skeletal tissue is a complex process that requires activation of adaptive mechanisms and a cross-talk between the tumor cells and the components of the metastatic niche^3^. One important feature of the bone marrow niche is that it is prone to oxidative and metabolic stresses. Within the bone marrow space, hypoxic and inflammatory events contribute to nutrient depletion, generation of reactive oxygen species (ROS) and subsequent mitochondrial damage^4^. Advanced age, obesity and various metabolic conditions that result in enhanced marrow adiposity are known to exacerbate stress conditions in the bone^5^. Marrow fat cells and elevated levels of fatty acids promote hypoxia signaling in the tumor cells^6^. They can also induce endoplasmic reticulum (ER) stress in both the tumor cells and the neighboring cells^7^. Although both ER stress and the oxidative stress pathways can be detrimental to the cell, they are often skillfully used by the tumor cells as mechanisms of overcoming the harsh microenvironmental conditions and surviving in the hypoxic niche^8,9^.

One key mechanism that tumor cells utilize to resist oxidative stress is the overexpression of heme oxygenase 1 (HO-1). HO-1 is an inducible enzyme, which in addition to its primary role in heme metabolism, has potent anti-oxidant properties and has been shown to mediate its effects through the reduction of ROS^10^. In normal cells, HO-1 plays cyto-protective roles by preventing oxidative injury, attenuating inflammatory responses and reducing cell death. In cancer, HO-1 overexpression has been associated with aggressiveness^10^ and its further upregulation in response to therapies has been linked to tumor resistance^11,12^. Notably, in a context of prostate cancer progression, the role of HO-1 has been controversial^11^. Similar to HO-1 expression patterns in several other types of cancers, HO-1 is robustly upregulated in prostate cancer patients with advanced disease as compared to healthy patients or patients with benign hyperplasia^13,14^. Silencing of HO-1 has been shown to inhibit tumor growth *in vitro* and *in vivo* ^13,15^. On the other hand, a decrease in prostate tumor cell proliferation and invasion, and inhibition of angiogenesis upon HO-1 upregulation, have been demonstrated in some experimental models^16,17^. This underscores the complex nature of this powerful enzyme and suggests that its tumor-regulatory functions might be context- and tumor site-specific.

Although HO-1 activity has been primarily associated with oxidative stress, it has also been shown to be protective against ER stress^18^. This is in line with growing evidence that oxidative stress and ER stress pathways are strongly interconnected, and that oxidative folding within the ER is a significant source of ROS^19,20^. To relieve ER stress, cells activate a transcriptional program called Unfolded Protein Response (UPR), orchestrated by the ER chaperone Glucose Regulated Protein 78 (GRP78; BIP)^21,22^, whose increased activity in response to ROS exposure protects the cells from oxidative damage^20^. BIP expression in clinical specimens has been correlated with tumor survival, chemoresistance and poor prognosis^23,24^, and its localization to the cell surface of prostate cancer cells has been linked to the activation of HO-1 regulator Nrf2, and resistance to ER-stress induced apoptosis^25^. HO-1 activity has also been linked to BIP activation in colon cancer cells, a process associated with increased invasiveness^26^. Both BIP and HO-1 are known to be induced by hypoxia^27,28^, but their roles in hypoxia tolerance and survival by metastatic tumor cells in bone have not yet been explored.

We have shown previously that HO-1 levels are induced in prostate cancer cells upon exposure to marrow adipocyte-secreted factors^29^. The goal of the present study was to investigate if upon induction of marrow adiposity, HO-1 expressed by the skeletal tumors protects them from oxidative stress and promotes survival. Through Oncomine database analyses, we demonstrate that HO-1 levels are significantly increased in human metastatic prostate cancer (PCa) tumors as compared to primary tumors. We also show that both HO-1 and BIP expression are increased in PCa cells interacting with marrow adipocytes *in vitro* and *in vivo*, a phenomenon that can be abrogated by an antioxidant treatment. Stable overexpression of HO-1 in PC3 and ARcaP(M) cells results in increased invasiveness *in vitro* and accelerated growth and progression in bone tumors *in vivo*. We further demonstrate that both adipocyte-induced oxidative stress and HO-1 overexpression result in splicing of the X-box protein XBP1 and activation of survival pathways involving survivin (BIRC5) and Bcl-xl in tumor cells. These findings offer new insight into mechanisms utilized by the metastatic cells in the bone microenvironment to gain survival advantage and thrive in the harsh metastatic niche.

## Results

### HO-1, BIP and XBP1 levels are induced in prostate cancer cells interacting with adipocytes

**Figure 1:**
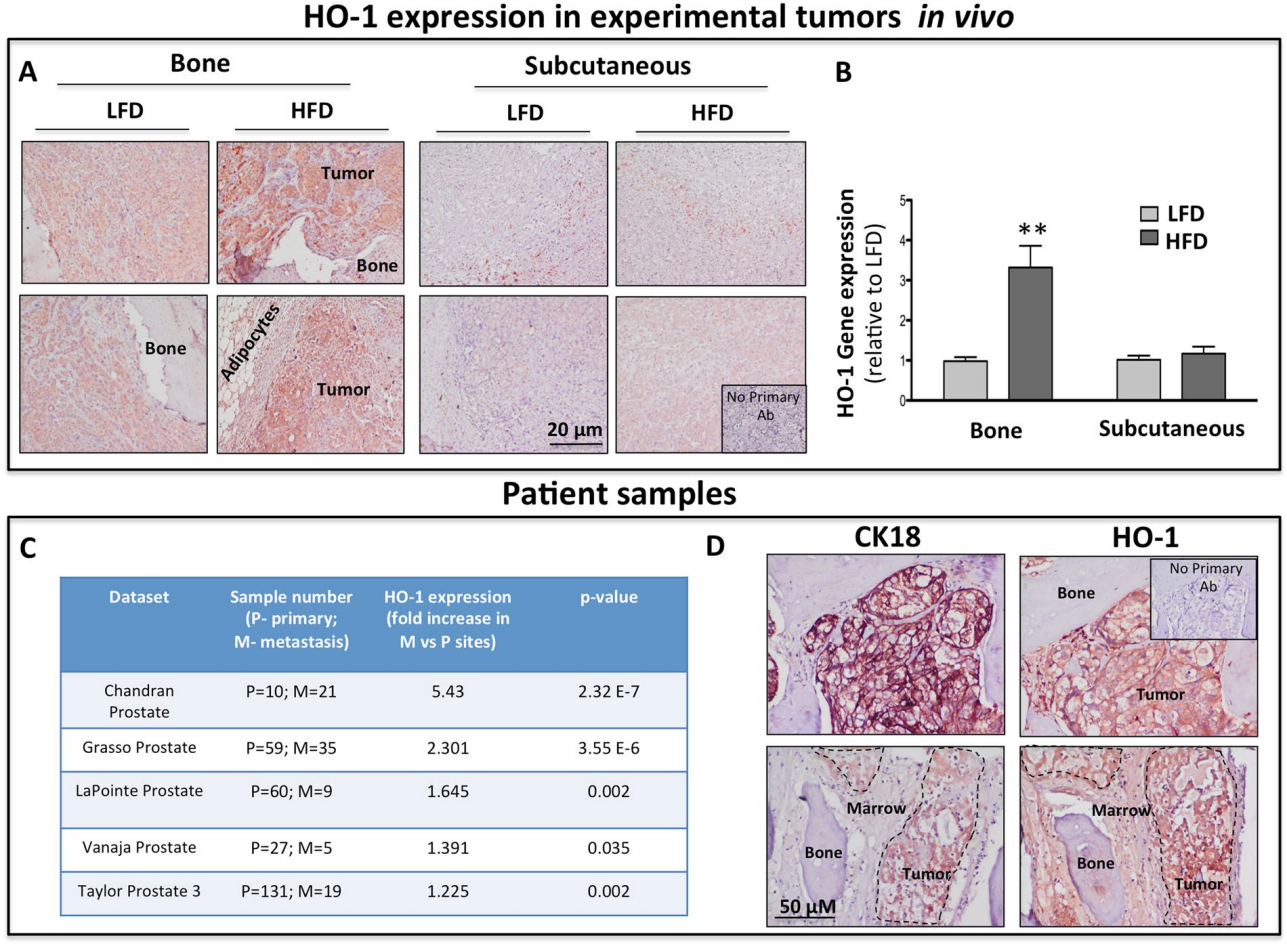
HO-1 is upregulated in experimental bone tumors and in metastatic tissues from prostate cancer patients. (**A**) Immunohistochemical analysis of HO-1 in intratibial (Left) and subcutaneous (Right) tumors from mice on low-fat diet vs high-fat diet. Itratibial HFD tumors show increased HO-1 staining. Serial sections without incubation with a primary antibody were used as a control (Inset). (**B**) HO-1 gene expression is increased in bone tumors but not subcutaneous tumors from mice with diet-induced obesity; Data are representative of 3 experiments. (**p < 0.01 is considered statistically significant). (**C**) Oncomine database analysis reveals significant upregulation of HO-1 in metastatic tumors compared to primary tumors in patients across five different databases. (**D**) Immunohistochemical analysis of cytokeratin-18 **(**CK18, Left**)** and HO-1 **(**Right**)** in bone core biopsies of metastatic prostate cancer tumors collected under KCI-approved protocol. CK18 was used as a control for tumor cell-specific marker in the bone microenvironment. Serial sections without incubation with a primary antibody were used as a control **(**Inset**)**.

It has been well-established that marrow adiposity is a major contributor to tumor progression in bone^30–33^. We have previously discovered that HO-1 is one of the most significantly upregulated genes in prostate bone tumors from mice with diet-induced marrow adiposity^29^. Upon further investigation, we also observed that the induction of this enzyme in response to high fat diet (HFD) occurs only in the intratibial and not in subcutaneous prostate tumors. Specifically, immunohistochemical detection of HO-1 protein clearly revealed increased HO-1 expression in tumor nests as opposed to surrounding stroma in bone tumors from HFD mice (Fig. 1A). This was further confirmed by increased HO-1 mRNA levels using human-specific Taqman RT PCR probes (Fig. 1B). To examine the relevance of HO-1 in human metastatic disease, we performed *in silico* Oncomine database analyses of several prostate datasets and determined that expression of this enzyme is higher in metastatic tissues than primary tumors from prostate cancer patients (Fig. 1C). Additional cBioPortal polyA transcriptome analysis of RNA-seq filtered for mRNA from the Metastatic Prostate Cancer, SU2C/PCF Dream Team cohort showed that across multiple metastatic tumor sites in patients there is strikingly high expression of HO-1 in bone lesions compared to other distal sites collected (Supplementary Figure 1). Importantly, our own immunohistochemical staining of bone biopsy tissues from metastatic patients confirmed high HO-1 presence in the cytokeratin 18-positive (CK18+) tumor cells colonizing the bone marrow (Fig. 1D). Although HO-1 is predominately expressed at high levels in CK18+ areas of the bone lesion, some HO-1 expression was detected in areas of the bone marrow not occupied by the tumor (Fig. 1D, bottom panels), which is consistent with the expression of this enzyme in normal cells^10,14,34^. However, the majority of HO-1 presence, and the highest intensity of staining were observed in CK18+ areas of the bone lesion. These novel findings prompted us to investigate the role of HO-1 in metastatic tumor cells in bone.

**Figure 2:**
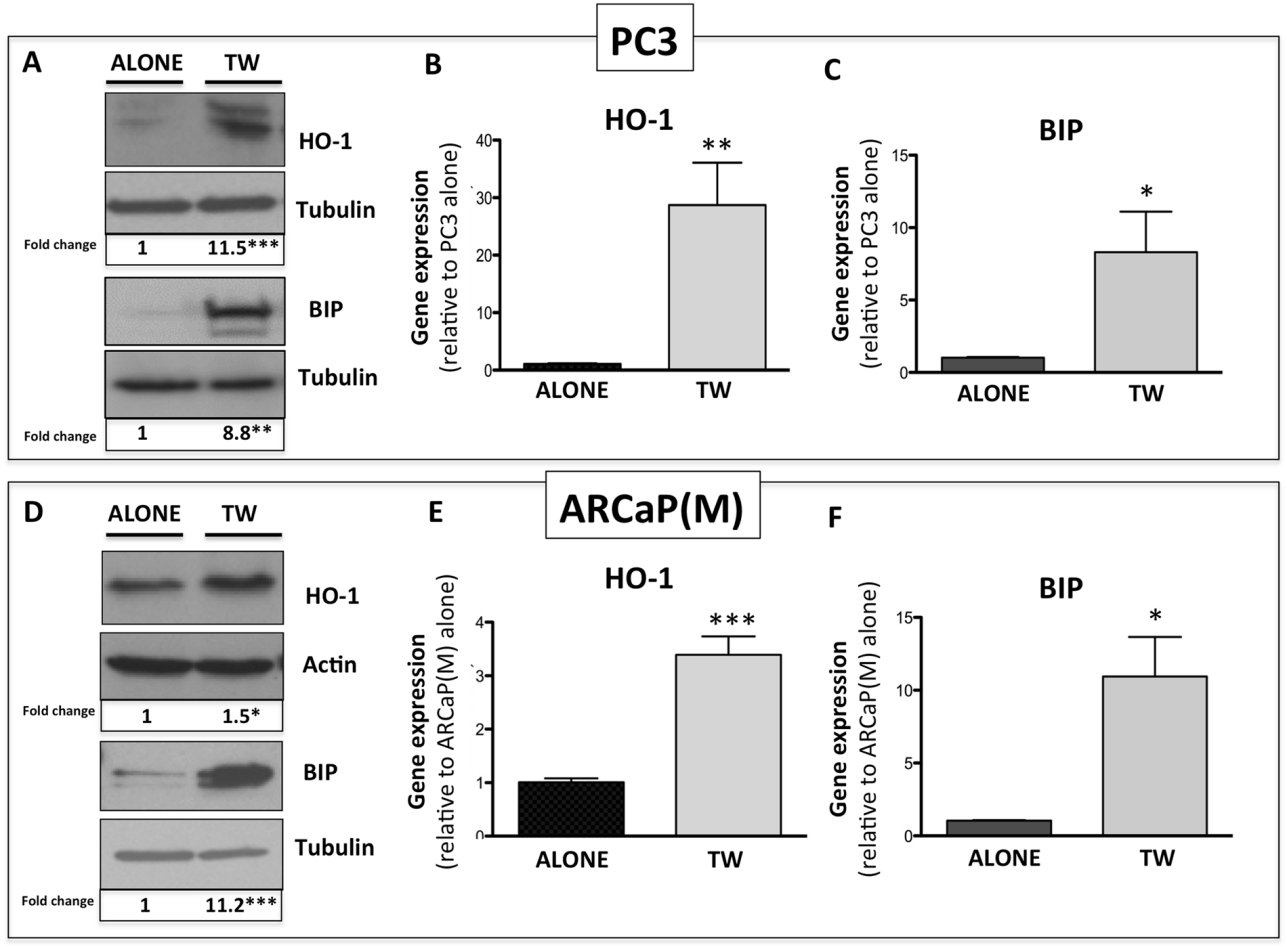
HO-1 and BIP are upregulated in PCa in response to adipocyte-derived factors. (**A**) Western blot analysis of HO-1 and BIP expression in PC3 cells alone or in transwell co-culture with bone marrow adipocytes; cropped blots from 2 independent gels; full size blots are included as Supplementary Figure 13. Taqman RT PCR analyses of HO-1 (**B**) and BIP (**C**) in PC3 cells cultured in the presence or absence of bone marrow adipocytes. (**D**) Western blot analysis of HO-1 and BIP in ARCaP(M) prostate cancer cells alone or in transwell co-culture with adipocytes; cropped blots from 2 independent gels; full size blots are included as Supplementary Figure 14. Gene expression analyses of HO-1 (**E**) and BIP (**F**) in ARCaP(M) cells exposed to adipocytes in transwell co-culture. RT-PCR analyses, normalized to HPRT1, were analyzed by DataAssist software. Tubulin (**A,D**) and β-Actin (**D**) were used as loading controls for western blot analyses and fold changes in protein expression based on densitometric analysis using ImageJ are provided below each band. Fold changes were calculated relative to tumor cells alone and blots shown are representative of 3 independent experiments. (***p < 0.001, **p < 0.01, and *p < 0.05 are considered statistically significant).

As our data clearly suggested a link between increased adipocyte numbers in bone and HO-1 expression in the skeletal tumor, we went on to determine if this is a direct effect of adipocyte exposure. We first examined HO-1 gene and protein levels in PC3 and ARCaP(M) cells grown in transwell co-cultures with marrow fat cells. Several-fold increases in HO-1 mRNA and protein levels were observed in tumor cells from transwell co-cultures as opposed to cells grown alone (Fig. 2A,B,D, and E). Interestingly, along with augmented HO-1 levels, there were highly prominent increases in the gene and protein expression of the ER chaperone BIP, a result suggesting an induction of ER stress (Fig. 2A,C,D, and F). This effect of bone marrow adipocyte-driven HO-1 and BIP induction was not specific to prostate cancer cells, as similar effects were observed in bone-seeking breast carcinoma MDA-MB-231BO cells (Supplementary Figure 2).

**Figure 3:**
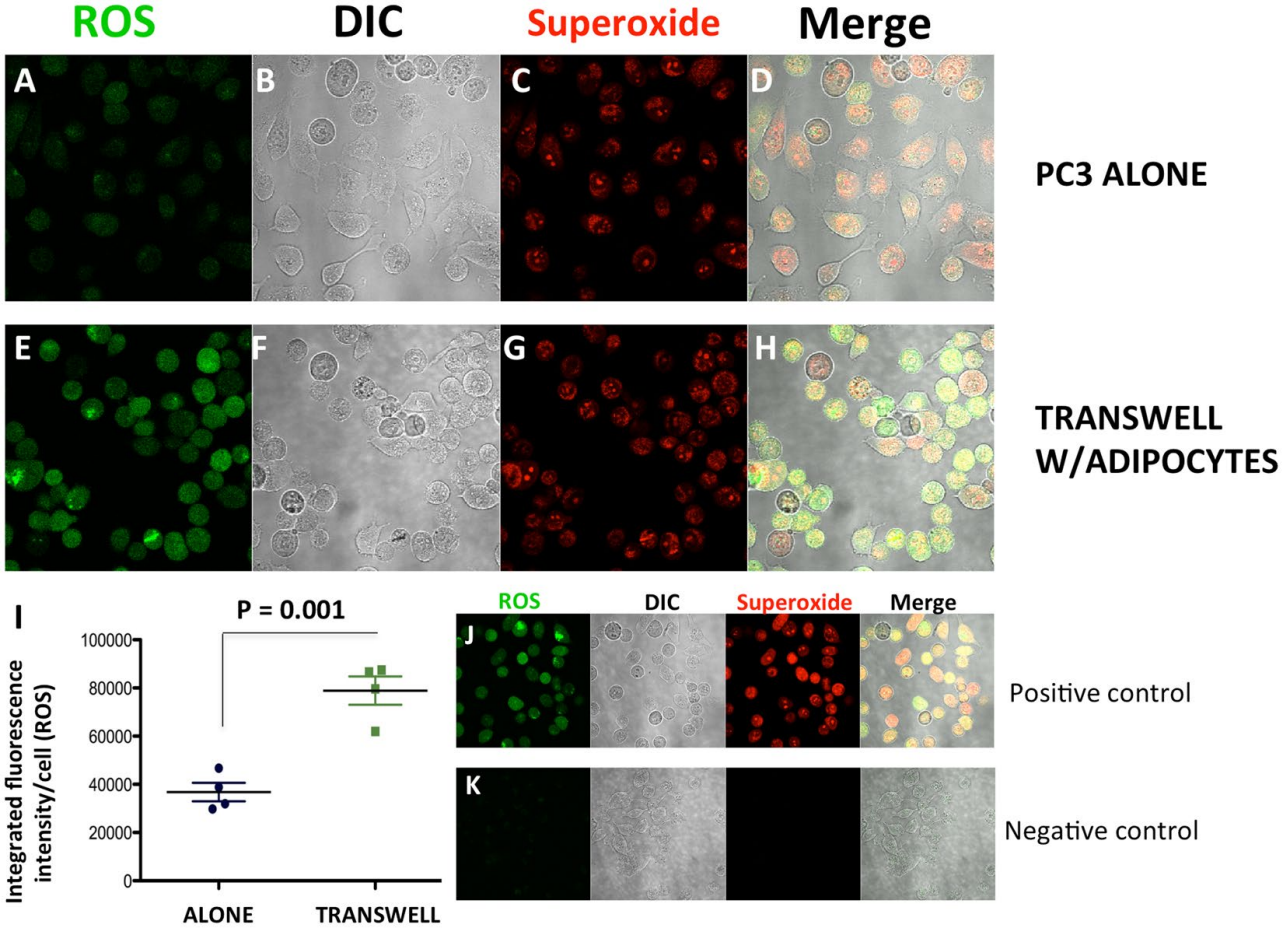
ROS accumulation in PCa cells increases with exposure to adipocytes. (**A**–**H**) ROS-ID^**®**^ Total ROS/Superoxide detection kit staining reactive oxygen species (ROS) (green, **A**,**E**) and superoxides (red, **C**,**G**), in control **(A**–**D**) or transwell (**E**–**H**) conditions using PC3 cells. DIC (**B**,**F**) and merged (**D**,**H**) images shown for overlay. (**I**) Integrated intensity of ROS per cell in alone vs transwell conditions. (**J**) Positive and (**K**) negative controls for the kit to ensure proper functionality.

**Figure 4:**
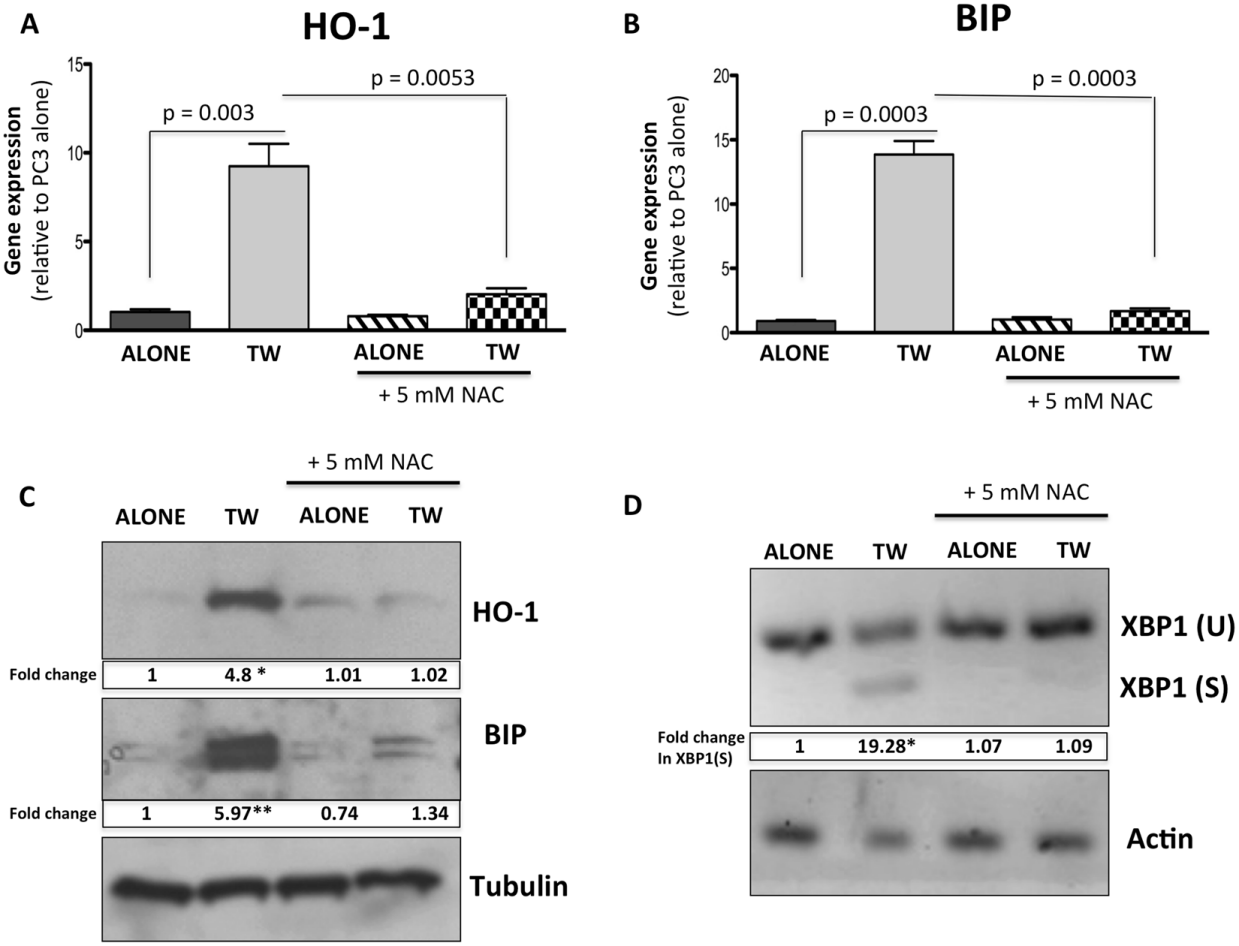
Stress response from adipocyte exposure can be negated with the addition of 5 mM NAC. (**A,B**) Taqman RT-PCR analysis of (**A**) HO-1 and (**B**) BIP in PC3 cells alone or in transwell co-culture with adipocytes, in the presence or absence of 5 mM N-acetyl-cysteine (NAC). (**C**) Western blot analysis of HO-1 and BIP in PC3 cells alone or in transwell co-culture with adipocytes, in the presence or absence of 5 mM NAC; cropped blots; full size blots are included as Supplementary Figure 15. (**D**) PCR analysis of spliced XBP1 in PC3 cells alone or in transwell co-culture with adipocytes, with or without 5 mM NAC. Taqman RT-PCR analyses were normalized to HPRT1. Tubulin was used as a loading control for protein expression, and Actin was used as a loading control for semi-quantitative PCR analysis. For both analyses fold changes in expression are provided below each band. Fold changes were calculated relative to tumor cells alone and blots shown are representative of 3 independent experiments. (**p < 0.01, and *p < 0.05 are considered statistically significant).

Since both HO-1 and BIP are important sensors of reactive oxygen species (ROS), and protectors from oxidative cell damage^12,20^, we next examined whether their induction upon interaction with adipocytes was associated with oxidative stress. We used a Total ROS/Superoxide detection kit, composed of a green-fluorescent dye to measure a wide range of reactive species, such as hydrogen peroxide, peroxynitrite, hydroxyl radicals, nitric oxide, and peroxy radicals, and an orange dye to detect superoxide. We determined that total ROS levels in tumor cells were significantly higher upon transwell co-culture with adipocytes (Fig. 3, green), suggesting adipocyte exposure induced an oxidative stress response in the tumor cells. Interestingly, there were no significant differences in the superoxide levels (red) between culture conditions, nor in the expression of superoxide dismutase (SOD2), one of the key enzymes responsible for superoxide regulation (Supplementary Figure 3). This suggests that reactive species other than superoxide (e.g., hydrogen peroxide, known as a primary ROS signaling molecule^35^), might be involved in adipocyte-induced oxidative stress. Treatment of the co-cultures with an antioxidant N-acetylcysteine (NAC) (Fig. 4A–C; Supplementary Figure 2) or glutathione (GSH; Supplementary Figure 4) brought the levels of both HO-1 and BIP back to baseline. In addition, both NAC and GSH significantly reduced adipocyte-induced ROS production in transwell co-cultures (Supplementary Figure 5). This further indicates that the expression of HO-1 and BIP is indeed likely regulated by the adipocyte-induced oxidative stress.

Notably, we also observed that adipocytes induce transcript levels and splicing of X-box Binding Protein 1 (XBP1), a factor governing UPR^36,37^, and this effect was abolished by the treatment with NAC (Fig. 4D). Interestingly, treatment of adipocyte-tumor cell co-cultures with Isoproterenol, an inducer of lipolysis, increased splicing of XBP1 and augmented BIP expression, indicating the importance of adipocyte-supplied lipids in ER stress induction in tumor cells (Supplementary Figure 6A). Accordingly, adipocyte-induced XBP1 splicing was reduced by the treatment with STF 083010 and MKC3946 (Supplementary Figure 6B,C), the inhibitors of XBP1-spicing enzyme IRE1^38,39^.

### Adipocyte-induced oxidative stress and HO-1 overexpression promote tumor cell invasion

**Figure 5:**
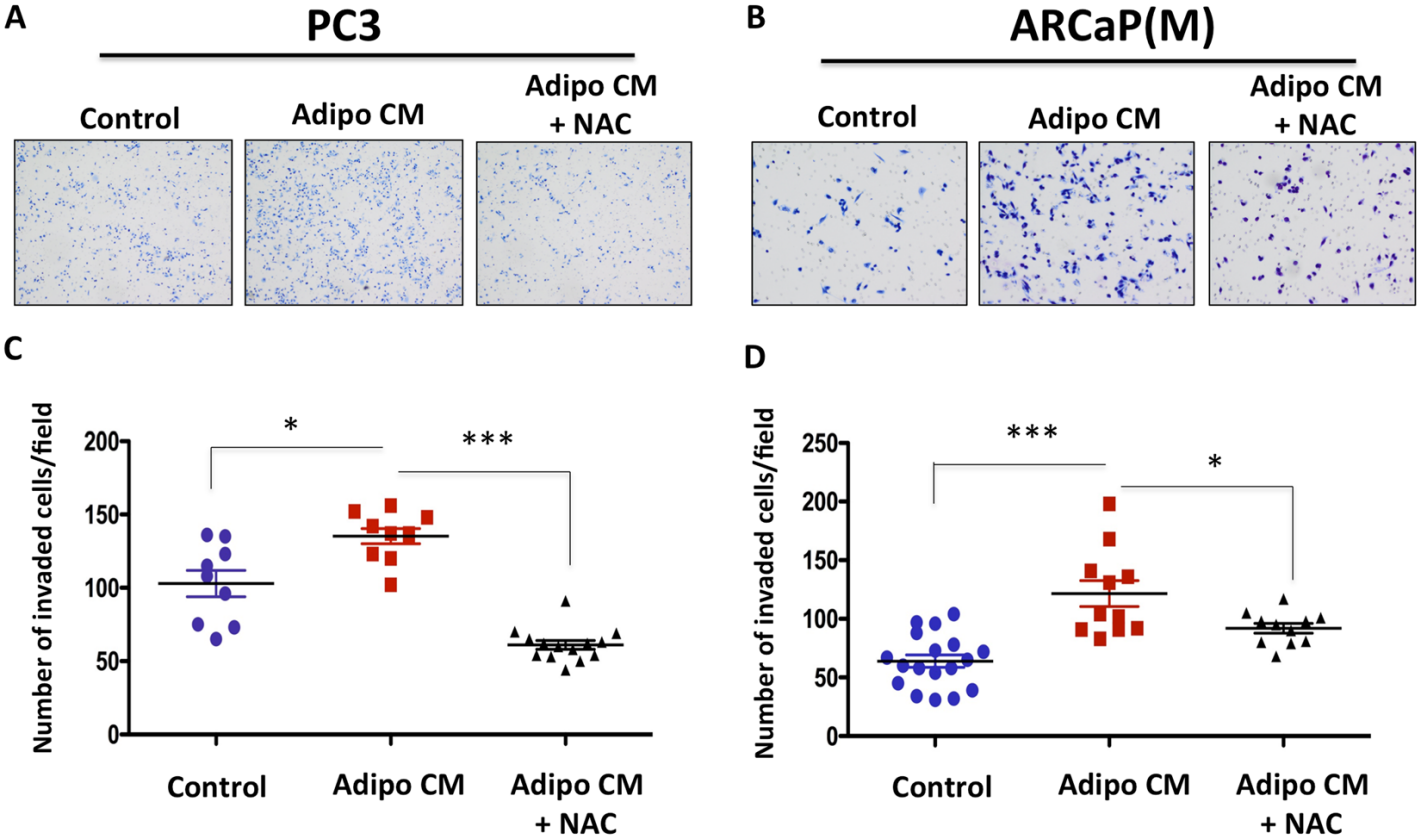
Adipocyte-induced invasion of PCa cells is inhibited by NAC treatment. (**A**) PC3 and (**B**) ARCaP(M) PCa were plated on rBM-coated filters in the absence or presence of adipocyte-conditoned meda (Adipo CM), with or without 5 mM NAC, and allowed to invade for 48 hr. Invaded cells were stained using Diff quick kit and counted. (**C,D**) Quantification of the number of cells that invaded upon exposure to Adipo CM, with or without 5 mM NAC. Data are shown as number of invaded cell/field for at least three experiments. (***p < 0.001 and *p < 0.05 are considered statistically significant).

We have reported previously that prostate and breast tumor cells exposed to adipocyte-derived factors are significantly more invasive than cells cultured under control conditions^29^. Since adipocyte-induced invasion appears to occur in parallel with the induction of oxidative stress and UPR, we speculated that NAC treatment would reverse this phenotype. Our analysis of PC3 and ARCaP(M) invasion through the reconstituted basement membrane (rBM) revealed significant reduction in the number of invaded cells with NAC treatment (Fig. 5), suggesting that adipocyte-induced oxidative stress promotes an invasive phenotype in prostate cancer cells. It is important to note that NAC treatment at the concentration used for this study did not significantly affect tumor cell viability as indicated by the Calcein A/M viability assay, Calcein staining and the absence of PARP cleavage (Supplementary Figure 7). This further confirms that the observed effects of NAC were invasion-specific.

**Figure 6:**
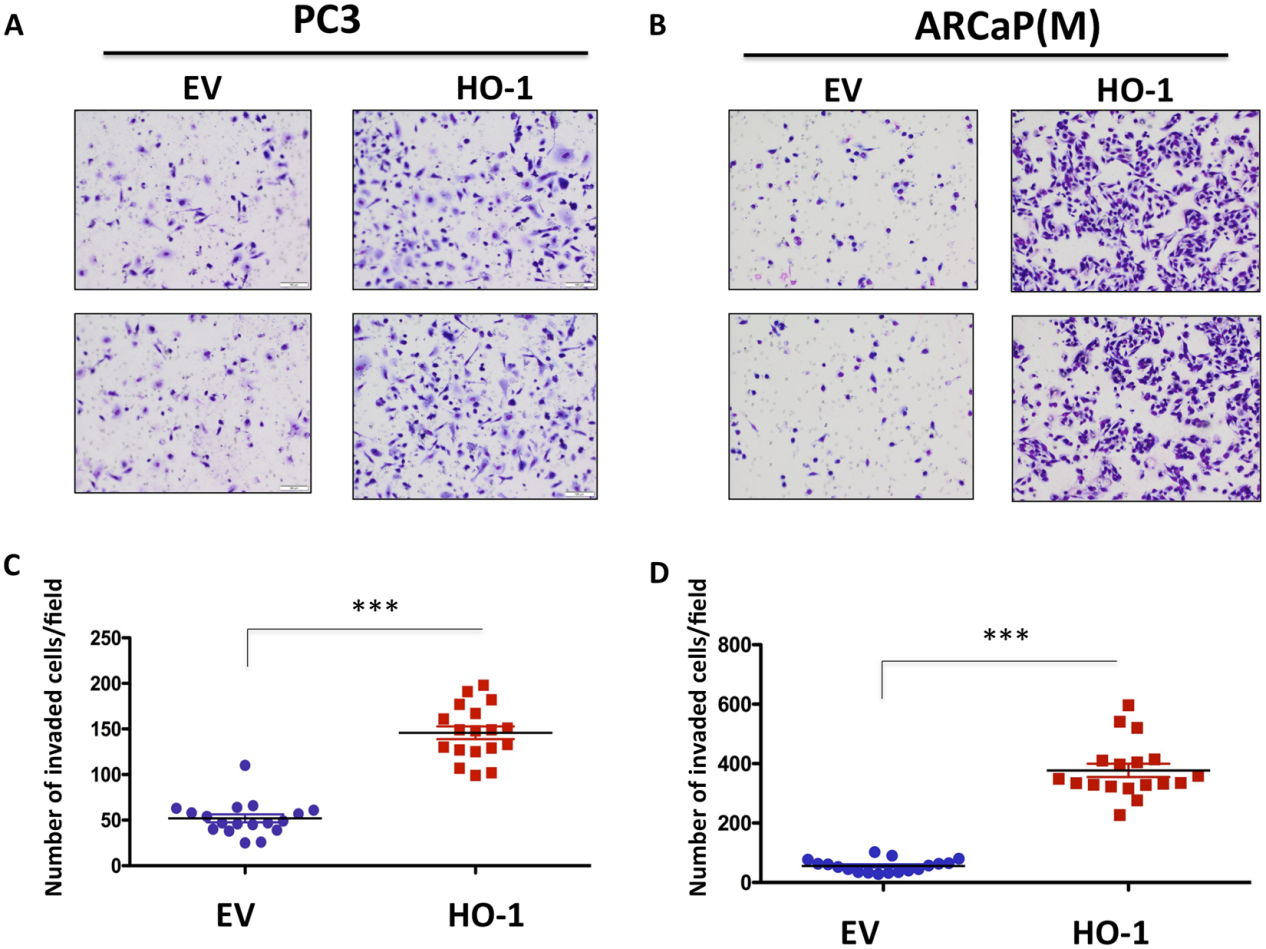
Overexpression of HO-1 in PCa cells increases the invasive potential *in vitro*. (**A**) PC3 and (**B**) ARCaP(M) PCa transfected with an empty vector or overexpressing HO-1 were plated on rBM-coated filters and allowed to invade for 48 hr. Invaded cells were stained using Diff quick kit and counted. (**C**,**D**) Quantification of the number of cells that invaded with or without overexpression of HO-1. Data are shown as number of invaded cell/field for at least three experiments. (***p < 0.001 is considered statistically significant).

Since HO-1 is one of the key antioxidant enzymes induced by oxidative stress and we have shown its expression is increased upon adipocyte exposure *in vitro* and *in vivo* (Fig. 1 and^29^), we reasoned that its stable overexpression in tumor cells in the absence of adipocytes would be sufficient to induce invasiveness. Indeed, both PC3 and ARCaP(M) cells stably overexpressing HO-1 (Supplementary Figure 8) were significantly more invasive than their empty vector counterparts (Fig. 6). This is consistent with previous reports linking HO-1 with invasive potential^10,40^.

### HO-1 overexpression promotes bone tumor growth *in vivo*

**Figure 7:**
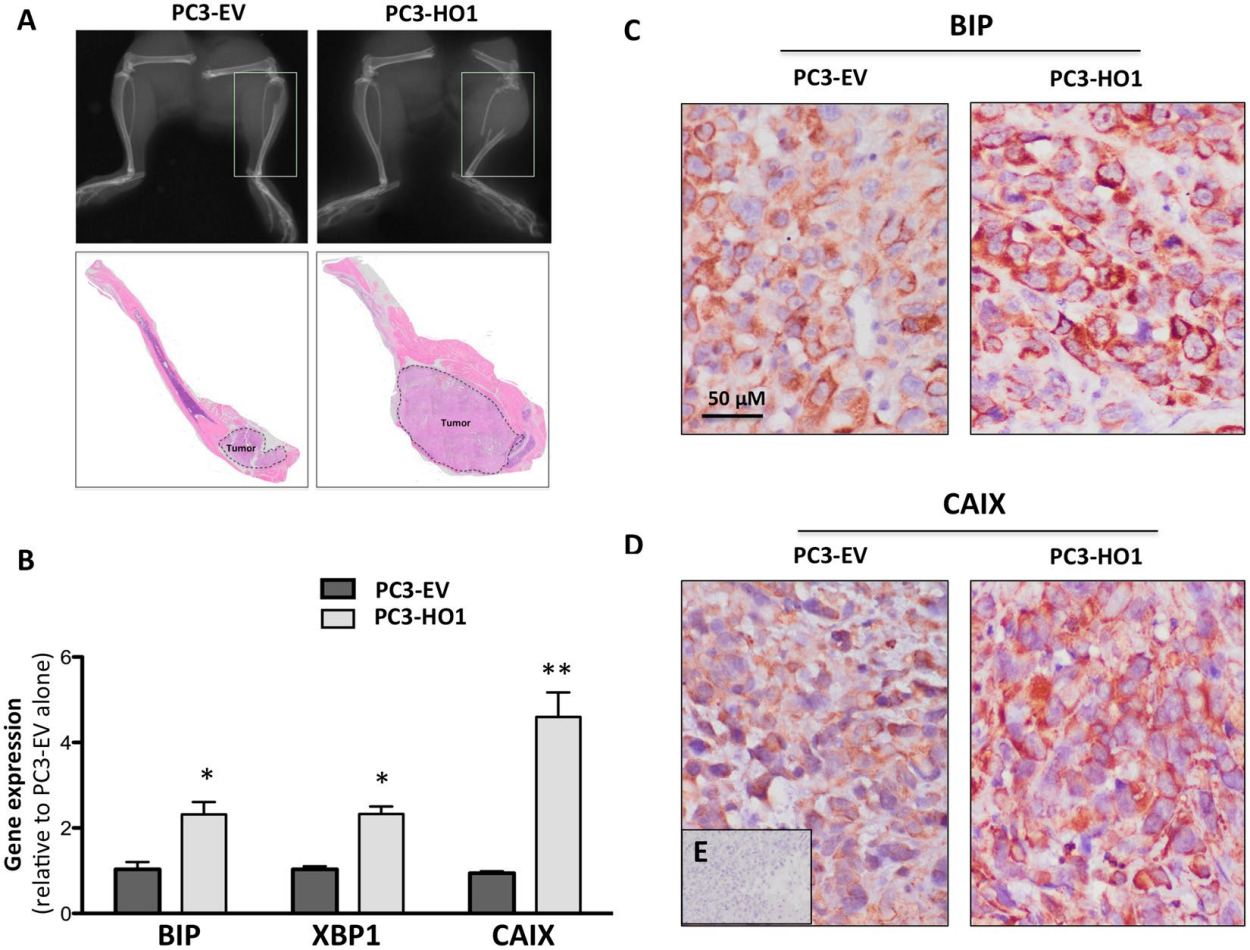
Overexpression of HO-1 in PCa cells increases tumor progression and hypoxic response *in vivo*. (**A**) X-ray and Hematoxylin and Eosin (H&E) staining of mouse tibia intratibially injected with PC3-EV or PC3-HO1 cells and allowed to grow for 6 weeks. (**B**) Taqman RT-PCR analysis of BIP, XBP1, and CAIX in tumor-bearing tibiae, comparing PC3-EV to PC3-HO1. (**C**) Immunohistochemical analysis of BIP and (**D**) CAIX in PC3-EV- vs PC3-HO1-bearing tibiae. (**E**) Serial section without incubation with a primary antibody was used as a negative control. RT-PCR analyses, normalized to HPRT1, were analyzed by DataAssist software. All images and PCR results are representative of at least three experiments. (**p < 0.01 and *p < 0.05 are considered statistically significant).

HO-1 effects on proliferation have been controversial, as both the pro- and anti-proliferative effects of this anti-oxidant enzyme on tumor cells have been reported^16,40–42^. Our analysis demonstrated that stable HO-1 overexpression had modest but significant proliferation-promoting effects on tumor cells *in vitro* (Supplementary Figure 9A). HO-1 overexpressing cells had increased evidence of XBP1 splicing, indicating activation of ER stress pathways (Supplementary Figure 9B). Notably, intratibial implantation of PC3 cells stably overexpressing HO-1 resulted in both significant increase in tumor growth and progression in bone (Fig. 7A). Similar effects on tumor growth and BIP expression were observed in ARCaP(M) tumors overexpressing HO-1 (Supplementary Figure 10). Taqman RT PCR analysis of PC3 bone tumors revealed significantly higher levels of BIP and XBP1 (Fig. 7B). Increased BIP expression with augmented HO-1 levels was also confirmed immunohistochemically in both PC3 and ARCaP(M) tumors (Fig. 7C and Supplementary Figure 10B).

Since HO-1 is known to be elevated under hypoxia^43^, and both HO-1 and XBP1 have been shown to regulate hypoxia-inducible factor 1-alpha (HIF-1α) in hypoxic tumor cells^44,45^, we next examined the levels of a direct HIF-1α target, carbonic anhydrase IX (CAIX), in response to HO-1 overexpression. CAIX transcript levels were significantly higher in HO-1 tumors as compared to EV tumors (Fig. 7B). These data are in agreement with the *in vitro* results of augmented CAIX and XBP1 levels in HO-1-overexpressing tumor cells, a phenomenon further potentiated by the interaction with adipocytes (Supplementary Figure 11). Immunohistochemical analyses of PC3 and ARCaP(M) tumors revealed typical membrane CAIX staining indicative of the hypoxic phenotype upon HO-1 overexpression (Fig. 7D and Supplementary Figure 10B).

### Exposure to marrow adipocyte and HO-1 overexpression promote tumor cell survival

**Figure 8:**
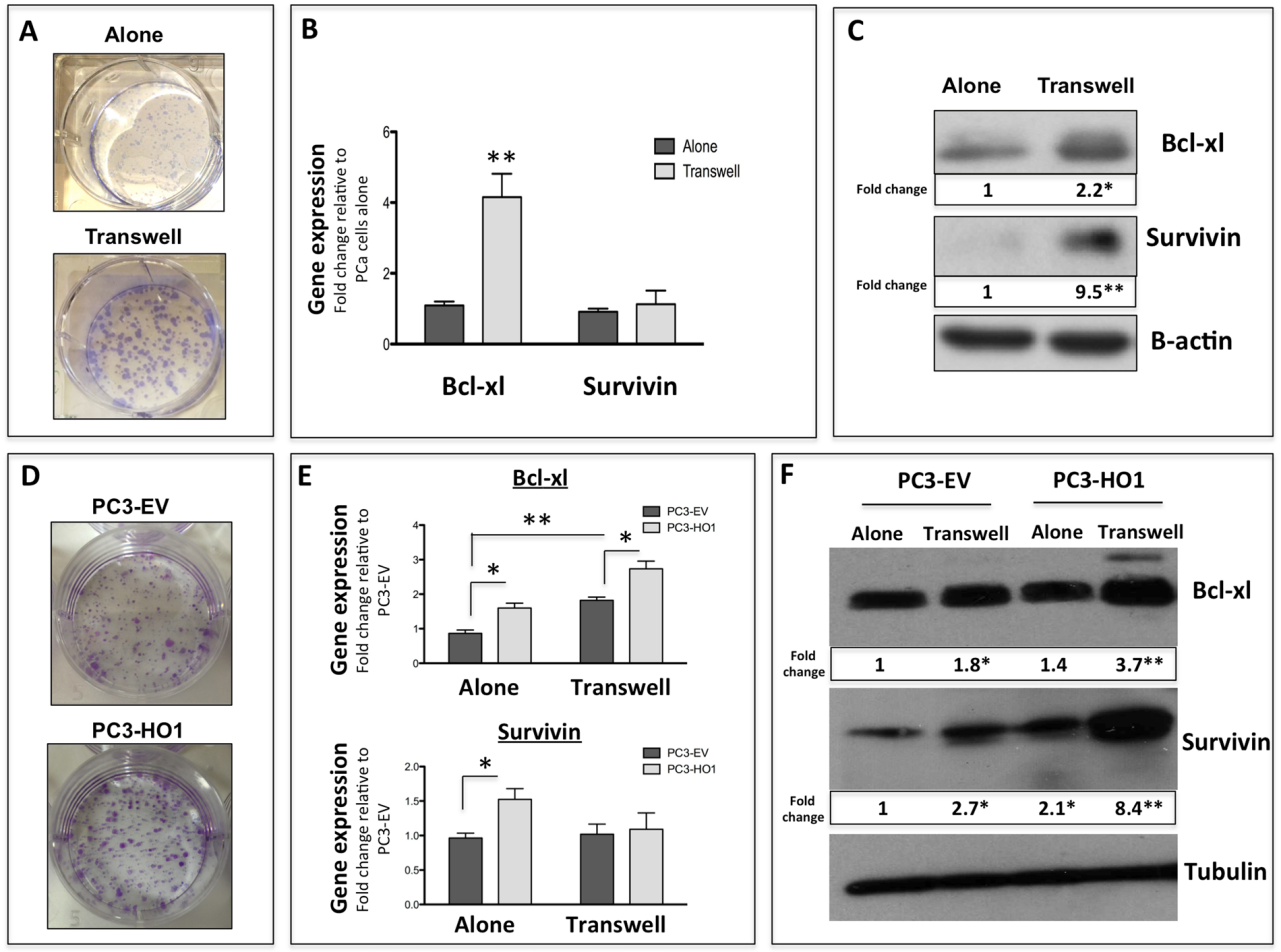
Survival of PCa is increased both with HO-1 overexpression and exposure to adipocyte-derived factors. (**A**) Clonogenic assay of PC3 cells either grown in control conditions or in transwell. (**B**) Taqman RT-PCR analysis and (**C**) Western blot analysis of Bcl-xl and Survivin of PC3 cells grown alone or in transwell; cropped blots from 2 independent gels; full size blots are included as Supplementary Figure 16. (**D**) Clonogenic assay of PC3-EV or PC3-HO1 cells. (**E**) Taqman RT-PCR analysis and (**F**) Western blot analysis of Bcl-xl and Survivin of PC3-EV or PC3-HO1 cells grown either in control or transwell conditions; cropped blots; full size blots are included as Supplementary Figure 17. Taqman RT-PCR analyses were normalized to HPRT1. β-Actin (**C**) and Tubulin (**F**) were used as a loading controls for protein expression and fold changes in protein expression based on densitometric analysis using ImageJ are provided below each band. Fold changes are shown relative to tumor cells alone for panel C and PC3-EV cells alone for panel F. The blots shown are representative of at least three experiments. (**p < 0.01 and *p < 0.05 are considered statistically significant).

Both oxidative stress and ER stress have been established as important mechanisms of cell survival and tumor progression^46–48^. To determine if exposure to adipocytes and subsequent induction of HO-1 and UPR promotes tumor cell survival, we cultured prostate tumor cells alone or in transwell with adipocytes, then performed a clonogenic assay. Tumor cells grown under transwell conditions developed significantly larger and more abundant colonies than the control cells (Fig. 8A). In addition, the expression of pro-survival factors Bcl-xl and survivin were significantly increased upon interaction with adipocytes (Fig. 8B,C), although survivin mRNA levels did not significantly change.

To determine if there is a direct link between HO-1 overexpression and activation of pro-survival mechanisms, we next examined clonogenic growth and gene and protein levels of Bcl-xl and survivin in PC3 cells overexpressing HO-1 and grown with or without adipocytes in transwell co-culture. Colonies formed by the PC3-HO1 cells were larger than those formed by the PC3-EV cells, indicating increased growth and survival despite the fact that the number of colonies did not differ significantly between the two cell lines (Fig. 8D). HO-1 overexpression alone had small but significant effect on Bcl-xl and survivin expression both at the gene and protein levels (Fig. 8E,F). Notably, interaction of HO-1 overexpressing cells with adipocytes led to robust upregulation of Bcl-xl and survivin protein, to levels that were significantly higher than those observed in EV cells exposed to adipocytes. This adipocyte-induced survivin protein expression was not, however, reflected by changes at the gene level, in agreement with data shown for the control transwell cultures in Fig. 8B. This suggests potential posttranscriptional regulation of survivin by HO-1, which is consistent with the literature evidence on survivin regulation^49^.

**Figure 9:**
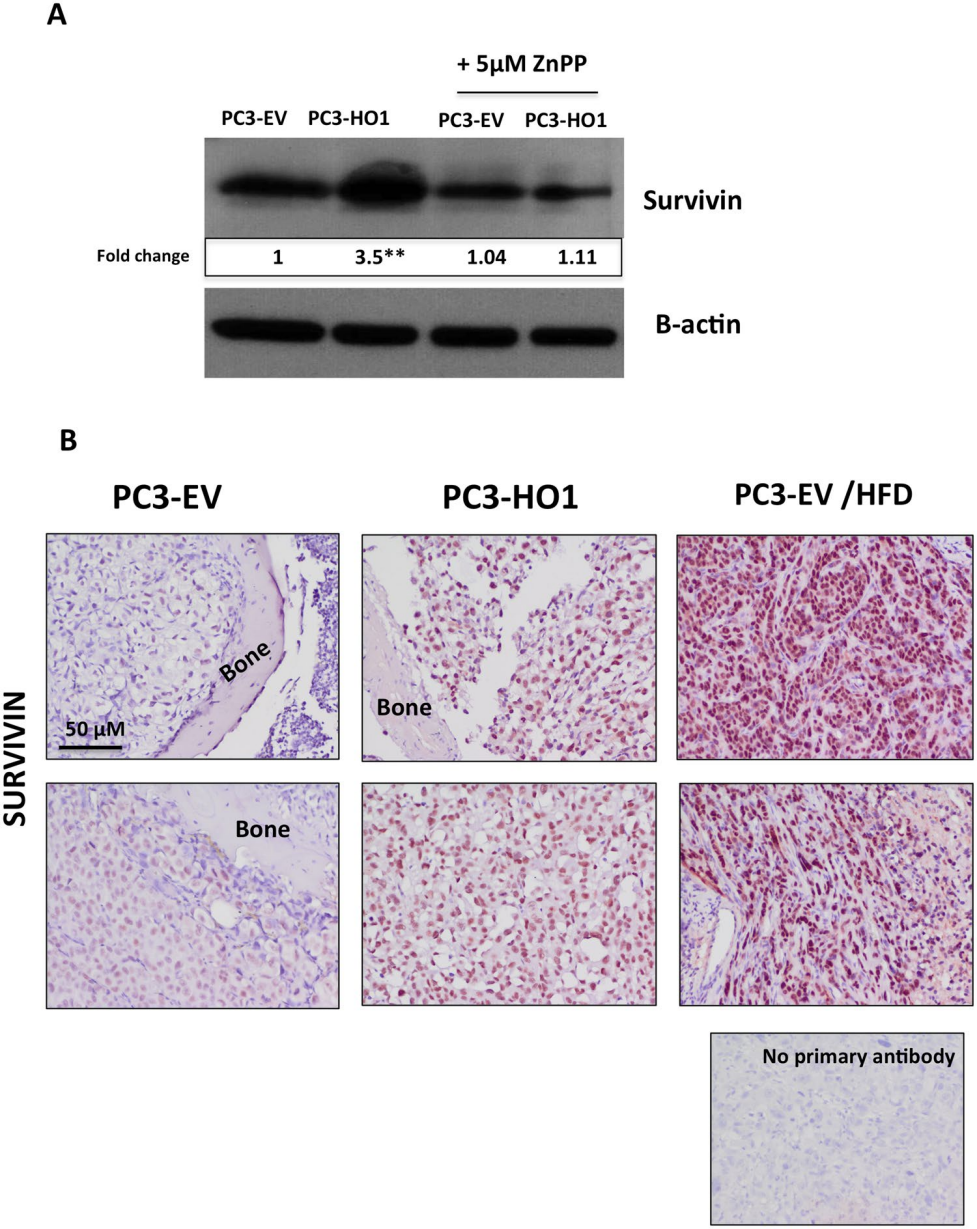
PCa survival is increased by HO-1 overexpression and the effects of high fat diet *in vivo*. (**A**) Western blot analysis of Survivin using PC3-EV or PC3-HO1 cells with or without 5 µM ZnPP; cropped blots; full size blots are included as Supplementary Figure 18; β-Actin was used as a loading control and fold changes in protein expression based on densitometric analysis using ImageJ are provided below each band. Fold changes are shown relative to PC3-EV cells alone and all results are representative of at least three experiments. (**p < 0.01 and *p < 0.05 are considered statistically significant). (**B**) Immunohistochemical analysis of Survivin in tumor-bearing tibiae from mice injected with PC3-EV (normal diet, left; high fat diet, right) or PC3-HO1 (middle). Section without primary antibody was used as negative control.

To examine whether HO-1-induced survivin expression is dependent on HO-1 activity, we treated PC3-EV and PC3-HO1 cells with zinc protoporphyrin IX (ZnPP), a known HO-1 inhibitor^50^. A robust increase in survivin levels, induced by HO-1 overexpression, was eliminated by the ZnPP treatment (Fig. 9A). Importantly, immunohistochemical analyses revealing increased survivin protein levels upon genetically-induced or HFD-induced HO-1 expression in prostate bone tumors *in vivo* (Fig. 9B) further underscored the importance of HO-1 in survivin regulation. This is of importance as survivin is highly expressed in prostate cancer, and its levels are particularly high in metastatic tissues from prostate cancer patients (Supplementary Figure 12).

## Discussion

Bone is a complex organ with a propensity to harbor metastatic lesions from a number of cancers, including prostate^1,2^. Despite being highly vascularized, bone tissue is very hypoxic, with oxygen concentrations generally ranging from 1–3%^51^, thus representing a uniquely harsh microenvironment for metastatic growth. Hypoxia signaling affects tumor progression by modulating tumor metabolism^6^, driving proliferation and survival, altering the behavior and phenotype of bone cells^52^, and allowing for selection of tumor cells capable of evading therapy^53^. Hypoxia is also one of the key inducers of oxidative stress and UPR^54–56^, and it is the resistance to oxidative stress and ER stress that allow malignant cells to turn on pro-survival signaling and thrive in the hostile microenvironments^57–59^.

Oxidative stress stems from the imbalance between ROS production and the cell’s ability to efficiently eliminate reactive species and repair the damages. It has been consistently postulated that persistently high ROS levels combined with the compensatory increases in antioxidant enzymes promote tumor progression by inducing DNA damage and genomic instability, as well as activating pro-survival signaling in the cell^60,61^. However, high levels of ROS can also be detrimental to cancer cells by making them susceptible to other stressors. Accordingly, it has been established that the pro- vs. anti-tumor functions of ROS molecules depend on the magnitude, persistence and site of their activation^60^. Both the approaches to induce ROS to toxic levels and the use of anti-oxidants have been explored therapeutically and a number of studies have shown the benefits of ROS inhibition in cancer therapy^60,62,63^. At the same time, several large clinical trials have failed to demonstrate significant therapeutic effects of antioxidants^62^. Some of those failures were attributed to non-specificity or poor bioavailability of antioxidant agents, others to the inhibition of ROS-mediated cell death^62^. Some recent laboratory studies have also suggested antioxidants can directly promote malignant transformation of cancer cells^64^ and are important for tumor initiation^65^, which adds to the controversy about the ROS involvement in cancer. However, given the undeniable evidence of ROS-mediated regulation of tumor cell proliferation^62,63,66^, invasion and metastasis^67,68^, as well as chemoresistance^69^ and the fact that ROS-mediated pathways in the bone tumor microenvironment are poorly understood, studies are needed to address the ROS paradox in bone-metastatic disease.

One of the cell types known to contribute significant amounts of ROS are adipocytes, especially under obese conditions^70^. ROS production has been shown to increase in parallel with fat accumulation in white adipose tissue adipocytes^71^. The increased ROS levels in adipocytes have been linked to mitochondrial dysfunction and metabolic pathologies^72^. Interestingly, an accumulation of fat in pathological bone marrow was implicated in ROS production due to fatty acid oxidation^73^. Notably, growing evidence also links oxidative stress and ROS production in bone marrow with skeletal aging and bone diseases^74,75^. However, there have not been studies to date investigating how bone marrow fat, so abundantly present in the aging or obese bone marrow^76–81^, might be contributing to the oxidative stress in the metastatic tumor.

The studies presented herein point to adipocyte-induced oxidative stress as a major contributor to tumor survival in bone. Our data show that upon exposure to adipocyte-rich environments *in vitro* or *in vivo*, prostate tumor cells upregulate the oxidative stress enzyme HO-1, a process that can be reversed by treatment with the anti-oxidant NAC. This is in line with well-established evidence that tumor cells are capable of developing sophisticated anti-oxidant systems to survive under high oxidative stress conditions^82,83^. Our data also demonstrate that HO-1 levels are highly induced in metastatic tissues from prostate cancer patients, and that forced expression of HO-1 in prostate tumor cells promotes tumor growth and invasion. It is important to point out that currently available Oncomine datasets do not distinguish bone metastases from other potential metastatic sites; therefore we can not explicitly state that HO-1 overexpression was restricted to bone tumors in those samples. However, given the strikingly high HO-1 expression in bone lesions revealed by cBioPortal analyses, the fact that our own immunohistochemical examination of bone tissues demonstrated high HO-1 levels in metastatic tumor cells, and the well-established evidence that more than 80% of metastatic prostate cancer patients present with bone lesions, the importance of this enzyme in bone-metastatic disease is clearly underscored.

Our findings presented herein support previous reports linking HO-1 overexpression with prostate tumor progression and aggressiveness^13–15^, while contrasting the studies that demonstrated an inverse correlation between HO-1 and tumor proliferation and invasion^16,17^. One reason for these differences in findings is the “double sword” nature of this inducible enzyme. The primary function of HO-1 is “antioxidant” protection via degradation of pro-oxidant heme and free radical scavenging^84^. However, under certain conditions, HO-1 activity and its downstream generation of iron and carbon monoxide can lead to ROS accumulation, creating “pro-oxidant” conditions^13,84^. This “pro-oxidant” function of HO-1 might be especially evident in the bone microenvironment, where uniquely hypoxic and harsh environmental conditions persist. A combination of hypoxia, abundance of fatty acids and lipotoxicity, as well as ER stress conditions, all come together to modulate the local redox environment and contribute to the increases in HO-1 activity, which, in a vicious cycle, further promotes oxidative stress. This indication of a bone-specific phenotype is supported by our finding of significantly induced HO-1 levels in prostate bone tumors, but not the subcutaneous tumors from mice with diet-induced obesity. Combined with patient data demonstrating high HO-1 presence in skeletal lesions, these results underscore the importance of HO-1 in metastatic disease that warrant further investigations. It is noteworthy that in addition to its enzymatic activity, HO-1 has been shown to be proteolytically cleaved and translocated to the nucleus, where it has been demonstrated to play a role in regulation of oxidant responsive transcription factors^85^. Indeed, our previous studies have shown that exposure to adipocyte-derived factors does promote nuclear localization of HO-1 in prostate tumor cells^86^. Although our present studies do reveal the importance of HO-1 activity in the regulation of survivin levels (Fig. 9), it is very likely that some of the pro-survival functions of the enzyme are independent of its activity. Ongoing studies in our laboratory are focusing on understanding the importance of both mechanisms in metastatic disease.

In addition to its involvement in oxidative stress regulation and protection, HO-1 has reported links to UPR and ER stress^18^. Indeed, our data reveal that co-incident with the induction of HO-1 upon adipocyte exposure, there is a significant escalation in the levels of ER chaperone BIP as well as increases in spliced XBP1, a finding indicating activation of UPR and linking oxidative stress and ER stress pathways in a context of adipocyte-driven tumor promotion. Our observation that both adipocyte exposure and HO-1 overexpression induce splicing of XBP1, a substrate of ER-localized transmembrane sensor IRE1^36^, is of importance. It is the spliced form of XBP1 that is known to act as a potent transcriptional regulator of UPR genes such as BIP, and a promoter of tumor cell survival^87^. Both XBP1 and its spliced form have been reported to be upregulated in several cancers, and the inhibition of IRE1/XBP1 pathway has been explored therapeutically^38,88^. Importantly, splicing and activation of XBP1 and its complex formation with HIF-1α was recently shown to enhance a hypoxic phenotype and subsequently promote the progression of triple negative breast cancers^45^. The XBP1/HIF-1α complex binds to and regulates genes associated with the Warburg phenotype^45^, and XBP1 silencing abrogates the glycolytic phenotype of gliomas^89^. We have shown previously that adipocytes promote oxygen-independent HIF-1α activation in prostate cancer cells and that HIF-1α signaling is responsible for adipocyte-induced glycolytic phenotype^6^. This suggests that adipocytes engage hypoxia and ER stress signaling pathways to support tumor growth in bone.

Another key finding from our study is that both stable overexpression of HO-1 and its induction by adipocytes result in increased levels of pro-survival factors, particularly survivin. Intriguingly, treatment of HO-1 overexpressing cells with HO-1 inhibitor ZnPP brings the survivin levels back to baseline, suggesting the importance of HO-1 activity in survivin regulation. Survivin is a member of the inhibitor of apoptosis protein (IAP) family, known to be regulated by HIF-1α, and its function is required to maintain cell viability under hypoxic conditions^90–92^. Our previous studies have established that HIF-1α is activated by adipocytes in prostate bone tumors^6^. Interestingly, one important feature of HIF-1α is that its stability can be regulated by carbon monoxide (CO) levels^93^, and CO is a product of HO-1 activity^10^. It is plausible that HO-1 overexpression, and subsequent increase in HO-1 activity, stabilize HIF-1α in tumor cells, driving downstream effects on pro-survival signaling. Our observed increases in levels of CAIX, a direct target of HIF-1α, upon HO-1 overexpression support this possibility. Survivin is expressed in almost all cancers and has been shown to be an important player in tumor aggressiveness and chemoresistance^94^. Our own Oncomine analysis of several prostate datasets revealed that its expression is particularly high in metastatic tissues as compared to primary tumors. A recent study in neuroblastoma revealed that survivin’s role in chemoresistance stems from its ability to shift tumor metabolism from oxidative phosphorylation to aerobic glycolysis^95^. This is highly relevant to bone metastatic tumors, which we have shown to undergo a metabolic switch to Warburg metabolism under conditions of increased marrow adiposity^6^. Understanding how these metabolic, ER stress, and survival pathways converge in a context of bone metastatic disease, and what the role of HO-1 is in survivin regulation, is of importance and warrants further investigations.

We focused our studies on prostate cancer because we have previously established the importance of marrow adiposity in growth and progression of these tumors in the skeleton^6,29,30,96^. However, our data in breast cancer bone-seeking cells MDA-MB-231BO cells suggest that this phenomenon extends beyond prostate cancer and may be relevant to other bone-trophic cancers. This could be of importance therapeutically, as all skeletal metastases share a common challenge of being incurable and difficult to treat. Our study identified HO-1 as an important driver of metastatic progression in bone. Inhibition of this enzyme as an approach to sensitize cancer cells to therapies has been extensively explored^12,13,97–99^ and has shown to be challenging, as HO-1 is capable of protecting tumor cells from death by both inhibiting apoptosis and autophagy induced by chemotherapeutic agents^42^. Several small molecule inhibitors have shown some success in increasing sensitivity to therapy in myeloid leukemias^100^, and some strategies in targeting HO-1 metabolites have also been proposed^12^, but more studies are needed to understand the therapeutic potential of HO-1 inhibition.

As evidence of multifaceted roles for HO-1 in cancer increasingly builds, there is a growing need for further exploration of molecular mechanisms behind the action of this powerful enzyme. Data presented herein uncover its involvement in adipocyte-driven regulation of pro-survival signaling in skeletal tumors. To our knowledge, this is the first study placing HO-1 at the cross-talk between oxidative stress, ER stress and survival pathways in metastatic progression in bone. Our findings identify survivin as a target of HO-1 and a mediator of adipocyte-induced survival in the metastatic niche. These findings have clinical implications, as survivin-overexpressing tumors have recently been shown to be sensitive to glycolysis inhibitors^95^. Understanding the molecular interactions between metabolic and stress pathways represents an important step towards improved treatment options for metastatic disease.

## Materials and Methods

### Materials

Delbecco’s modified Eagle’s medium (DMEM), RPMI-1640 medium (RPMI), minimum essential medium (MEMα), Protoporphytin IX zinc(II) (ZnPP), glutathione, isoproterenol, and other chemicals, unless otherwise stated, were obtained from Sigma Aldrich (St. Louis, MO, USA). HyClone fetal bovine serum (FBS) was from ThermoFisher (Pittsburg, PA, USA). Trypsin-EDTA, Alexa Fluor 488-conjugated goat anti-rat and anti-rabbit IgG, Hoechst Dye, and Gentamicin (G418) were from Invitrogen (Carlsbad, CA, USA). StemXVivo Adipogenic Supplement and goat anti-human/mouse heme oxygenase (HO-1) was from R&D Systems (Minneapolis, MN, USA). Rosiglitazone and *N*-acetylcysteine (NAC) was from Cayman Chemical Company (Ann Arbor, MI, USA). ROS-ID Total ROS detection kit was from Enzo (Farmingdale, NY, USA). PureCol collagen type I was from Advanced Biomatrix (San Diego, CA, USA). Cultrex™ (rBM; reduced growth factor) was from Trevigen (Gaithersburg, MD, USA). Transwell systems (Costar™ Transwell™ Permeable Supports with 0.4 µm pore size) and Falcon™ Cell Culture Inserts (invasion chambers with 8.0 µm pore size) were from Corning (Corning, NY, USA). RNeasy Mini Kits, RNeasy Plus Mini Kits, and QIAshredders were from Qiagen (Valencia, CA, USA). Immunoblotting Luminata Forte Western HRP substrate was from EMD Millipore (Billerica, MA, USA). Z-Fix was from Anatech, LTD (Battle Creek, MI, USA). Rabbit anti-human BIP, rabbit anti-human Bcl-xl, rabbit anti-human Survivin, rabbit anti-human PARP, and rabbit anti-human XBP-1s were from Cell Signaling Technologies (Danvers, MA, USA), and rabbit monoclonal carbonic anhydrase 9 (CAIX) was from Abcam (Cambridge, MA, USA). Mouse anti-human tubulin was from Developmental Studies Hybridoma Bank. University of Iowa, and mouse anti-human cytokeratin 18 (CK18) was from Dako (Utrecht, Netherlands). STF-083010 and MKC-3946 were from Calbiochem (San Diego, CA, USA).

### Cell Lines

PC3 cells, an androgen-independent osteolytic cell line derived from a bone metastasis of a high-grade adenocarcinoma, were purchased from American Type Culture Collection (ATCC; Manassas, VA, USA). ARCaP(M) cells, an androgen-repressed metastatic prostate cancer M (‘Mesenchymal’ clone) cells, were purchased from Novicure Biotechnology (Birmingham, AL, USA). Both lines were stably transfected with empty vector (E) and HO-1 (H) via myc-DDK vector plasmids (OriGene, Rockville, MD), which contain the neomycin-resistant gene. Transfection was performed using Lipofectamine 2000 and stable clones were selected, expanded, and maintained in medium supplemented with G418 (400 µg/ml for PC3, 800 µg/ml for ARCaP(M)). The MDA-MB-231BO is a bone-seeking clone derived from MDA-MB-231 breast carcinoma cells and was kindly provided by Dr. Toshiyuki Yoneda (University of Texas Health Science Center, San Antonio, TX). PC3 and MDA-MB-231BO cells were cultured in Dulbecco’s modified Eagle’s medium (DMEM) supplemented with 10% FBS, 10 mM HEPES, and 100 U/ml penicillin-streptomycin. ARCaP(M) cells were cultured in RPMI-1640 medium supplemented with 5% FBS, 10 mM HEPES, and 100 U/ml penicillin-streptomycin. Human cell lines have been authenticated by by the WSU Genomics facility.

Primary mouse bone marrow stromal cells (mBMSC) were isolated from tibiae and femurs of 6- to 8-week old FVB/N mice according to previously established protocols. To induce bone marrow adipocyte differentiation, mBMSCs were plated in 3D Collagen I gels, grown to confluency for 48–72 h, and treated with adipogenic cocktail (30% StemXVivo Adipogenic Suppliment, 1 µM insulin, 2 µM Rosiglitazone; DMEM and 10% FBS) for 8–10 days as previously described. Differentiated bone marrow adipocyte cultures were washed 3 times with PBS and used in experiments, or serum-starved overnight for collection of adipocyte-conditioned media (Adipo CM). All cells were maintained in a 37 °C humidified incubator ventilated with 5% CO_2_ and were routinely tested for the presence of mycoplasma.

### Animals

All experiments involving mice were performed in accordance with the protocol approved by the institutional Animal Investigational Committee of Wayne State University and NIH guidelines. *In vivo* xenograft studies were performed in 8- to 10-week old male mice in the FVB/N background with homozygous null mutation in the Rag-1 gene (FVB/N/N5, Rag-1^−/−^). All mice were bred in house.

### Clinical specimens

Bone biopsy tissue specimens were obtained from prostate cancer patients enrolled in human protocol # 2011–185 and approved by Karmanos Cancer Institute and Wayne State University Institutional Review Board. Written informed consent was obtained from all patients participating in the study and all immunohistochemical analyses were performed according to the procedures approved by the protocol and in agreement with protocol guidelines and regulations. Additional samples for validation analyses were obtained commercially through US Biomax (array # PR956).

### Intratibial injection of prostate cancer cells

Intratibial tumor injections were performed under isoflurane inhalation anesthesia according to previously published procedures^29,101^. Briefly, a cell suspension containing 5 × 10^5^ of PC3/ARCaP(M) cells in PBS (20 µl, right tibia), or PBS alone (control, 20 µl, left tibia) was injected into the bone marrow. Mice were euthanized six weeks (PC3 cells) or eight weeks (ARCaP(M) cells) post-injection, and control and tumor-bearing tibiae were removed. The bones were then imaged via x-ray using a Carestream *In Vivo* Xtreme Imager (Carestream, Rochester, NY). Approximately half of the tibiae samples from each group of PC3-bearing mice and one quarter of the tibiae samples from each group of ARCaP(M)-bearing mice were fixed in Z-fix, decalcified, and embedded in paraffin. The remaining samples were snap-frozen in liquid nitrogen, powderized using a tissue pulverizer, and stored at −80 °C for RNA analyses, completed using Trizol and the protocol from RNeasy Mini Kit.

### Bone histomorphometry and tumor size

Longitudinal sections (5 µm thick) from the control and tumor-bearing tibiae were deparaffinized and stained with hematoxylin and eosin, as described previously^29,101^. Digital images were captured under 4x magnification using an Olympus BX43 upright light microscope with an Olympus UC50 (CCD chip) camera (Olympus Scientific Solutions, Waltham, MA). The entire area of each tibia was reconstructed from the 4x images.

### Immunohistochemistry

Longitudinal sections (5 µm thick) from the control and tumor-bearing tibiae were deparaffinized and examined by immunohistochemistry for expression and localization of BIP (1:200), survivin (1:100), and CAIX (1:50). Human bone biopsy samples were decalcified, paraffin embedded, deparafinized, and cut into 5 µm sections. Immunohistochemical staining was performed to determine expression of localization of CK18 (1:50) and HO-1(1:100). For both xenograft and patient samples ImmPRESS Anti-Goat Peroxidase Polymer Detection systems along with a NovaRED kit (Vector Labs, Burlingame, CA) as a substrate were used for the peroxidase-mediated immunostaining reaction.

### Transwell co-culture

The mBMSC cells were embedded in Collagen, plated in 6-well plates and differentiated into adipocytes according to our previously published protocols^6,29^. Briefly, collagen-embedded mBMSCs were grown to confluency for 48–72 hours and treated with adipogenic cocktail (30% StemXVivo Adipogenic Suppliment, 1 μM insulin, 2 μM Rosiglitazone; DMEM and 10% FBS) for 8–10 days. After mature adipocyte cultures were established, tumor cells were seeded on top of a Transwell filter (0.2 µm pore size) to allow sharing of soluble factors between the two cell types. After 48-hour co-culture, cells were washed with PBS and collected for RNA and protein analyses. For RNA extraction, tumor cells were trypsinized, and adipocytes were dissociated from collagen using 0.1% collagenase. Both cell types were then collected into RLT Plus buffer. RNA was purified following the protocol in the RNeasy Plus Mini Kit. For protein collection, cells were washed three times with PBS, trypsinized, and lysates re-suspended in SME buffer with protease (MBL International, Woburn, MA) and phophatase (Thermo Scientific, Waltham, MA) inhibitors.

### ROS/Superoxide Staining

Tumor cells were plated on Cultrex-coated coverslips (thin layer only) in a 24-well plate at a density of 50,000/coverslip. Cells were given several hours to attach to coverslips, then transferred to the top of a Transwell filter over adipocytes (Transwell) or into an empty 6-well plate (Control) (2 coverslips/well), making sure there were extra coverslips for positive and negative controls. Cells were incubated for a total of 48 hours prior to imaging. The night before imaging, half of the wells had 5 mM NAC added to inhibit reactive oxygen species (ROS). The day of imaging, wells were changed to fresh media. A negative control coverslip was treated with 5 mM NAC for 30 minutes prior to induction. Cells were then incubated for an hour at 37 °C with the induction solution - 2x ROS Detection Solution in phenol red-free media using Oxidative Stress Detection Reagent and Superoxide Detection Reagent at a final concentration of 2.5 µM each. For positive control, pyocyanin, a ROS inducer, was added to one of the coverslips. The coverslips were washed 2x with provided wash buffer, and images were captured with a Zeiss LSM 780 confocal microscope (Carl Zeiss AG, Göttingen, Germany) using a 40x immersion lens. Oxidative stress was captured using excitation of 490 nm and emission of 525; Superoxide detection was captured using excitation of 550 nm and emission of 620 nm.

### Cell Viability Assays

#### MTT Assay

Vybrant® MTT cell proliferation assay (Life Technologies) was used to determine the difference in tumor cell proliferation. Clones were seeded in a clear 96-well plate at a density of 5,000 cells/well in 200 µl growth media. After 48 hours of incubation, the conversion of MTT (3-(4,5-dimethylthiazol-2-yl)-2,5-diphenyltetrazolium bromide) to formazan by viable tumor cells was measured at 540 nm according to manufacturer’s instructions.

#### Calcein AM Assay

Calcein AM Assay (Trevigen) was used to assess cell viability in the presence of increasing concentrations of NAC. Cells were seeded in black-walled 96-well plates at a density of 5,000 cells/well, along with NAC, in 200 µl growth media. After 48 hours of incubation, calcein AM was applied per manufacturer’s instructions, and the plate was read at an excitation of 490 nm and emission of 520 nm.

#### Calcein AM Cell Staining

Calcein AM cell staining was preformed to optically validate the viability of cells grown under NAC conditions. Cells were seeded on coverslips in 24-well plates at a density of 50,000 cells/coverslip, along with NAC, in 1 ml growth media. To stress cells, 0.4 µM Staurosporine was added overnight. After 48 hours of incubation, cells were stained with 2 µM Calcein AM in PBS at room temperature for 30 minutes, then gently washed and imaged on a Zeiss LSM 780 confocal microscope using excitation of 488 nm and emission of 515 nm.

### Immunoblot analyses

Lysate and media samples were loaded based on DNA concentrations in the corresponding lysates and proteins were electrophoresed on 12% or 15% SDS-PAGE gels, transferred to PVDF membranes (Bio-Rad, Hercules, CA, USA) and immunoblotted for HO-1, BIP, Bcl-xl, Survivin, XBP1s, Tubulin (1:2000), PARP, and β-actin (1:1000). All horseradish peroxidase-labeled secondary antibodies were used at 1:10,000. All images are in compliance with the digital image and integrity policies. For cropped blots, full size images are included as part of supplementary material.

### TaqMan RT-PCR

The cDNA from cells and *in vivo* samples was prepared from 1–2 µg of total RNA using High-Capacity cDNA Reverse Transcription kit (Applied Biosystems, Foster City, CA, USA). The analyses of genes were performed using TaqMan® Individual Gene Expression assays for Human HO-1 (Hs01110250), BIP (Hs00607129), CAIX (Hs00154208), Bcl-xl (Hs00236329), BIRC5/survivin (Hs04194392), SOD2 (Hs00167309), Epcam (Hs00901885), and XBP1 (Hs00231936). Assays were done on three biological replicates using TaqMan® Fast Universal PCR Master Mix and 50 ng of cDNA/well. All reactions were run on an Applied Biosystems StepOnePlus™ system. Three biological replicates of each sample were pooled together and assays were run in at least triplicate. All data were normalized to hypoxanthine phosphoribosyltransferase (HPRT1; Hs02800695) or 18 S (Hs03003631). DataAssist™ Software (Applied Biosystems) was used for all analyses..

### XBP-1 splicing PCR

PCR was used to evaluate the relative expression levels of XBP-1 splicing. Human XBP-1 primer sequences are; 5′-CCTGGTTGCTGAAGAGGAGG-3′ and 5′-CCATGGGGAGATGTTCTGGAG-3′. β-actin was used as the loading control with the primer sequence; 5′-GGATGCAGAAGGAGATCACTG-3′ and 5′-CGATCCACACGGAGTACTTG-3′ (primers from Integrated DNA Technologies, Coralville, IA, USA). PCR products were run on 4% Agarose gels. PCR products were imaged using Luminescent Image Analyzer LAS-1000 Plus.

### Invasion assays

Tumor cells were serum starved overnight and seeded on top of the BD invasion filter (8 µm pore size) coated with 3-D Culture Matrix™ reduced growth factor basement membrane extract (0.1 mg/ml). Cells were seeded at the density of 1 × 10^5^ cells/filter in serum-free media. Media containing the normal growth amount of serum (10% FBS for PC3 cells, 5% FBS for ARCaP(M) cells) was added to the bottom chamber as a chemoattractant. Cells were allowed to invade for 24 hours and then filters were fixed and stained with Kwik-Diff Staining reagents (Thermo Scientific). Invaded cells were visualized and captured using an Olympus BX43 upright light microscope under 10x magnification, and were manually counted using ImageJ software (National Institutes of Health, Bethesda, MD). Data were collected from at least three independent experiments preformed in triplicate.

### Clonogenic assays

Tumor cells were grown in alone or transwell conditions with adipocytes for 48 hours, then harvested with 0.25% Trypsin-EDTA and counted. Cells were plated in a 6-well plate at a concentration of 400 cells/well in regular growth media and allowed to grow for 14 days, with media replenishment occurring after 7 days. Cells were then fixed with cold methanol and stained with 0.1% Crystal Violet at room temperature. Stain was washed off with tap water and images were captured using a cell phone camera.

### Oncomine analyses

The Oncomine database (Oncomine™ v4.5: 729 datasets, 91,866 samples) was used for the analysis of primary (P) vs. metastatic (M) tumors by employing filters for selection of conditions and genes of interest (prostate cancer; metastasis vs. primary; genes). Data were ordered by ‘overexpression’ and the threshold was adjusted to P-value < 1E^−^4; fold change, 2; and gene rank, top 10%. For each database, only genes that met the criteria for significance were reported.

### cBioPortal analysis

The cBioPortal for Cancer Genomics^102,103^ was utilized to assess *HMOX1* mRNA expression in patient metastatic prostate tumors. Using the Metastatic Prostate Cancer, SU2C/PCF Dream Team cohort^104^, we performed a query for *HMOX1*. We then plotted *HMOX1* mRNA expression against the clinical attributes “Sample Type” and “Tumor Site”, allowing us to examine the levels of *HMOX1* expression (Y Axis) across metastatic prostate cancer patients presenting in various metastatic sites, including bone (X Axis). mRNA was isolated in this cohort by PolyA selection and data are shown as log2 scaled analysis of RNA Seq Reads per Kilobase Million (RPKM).

### Statistical analyses

For all analyses, data were presented as mean of at least 3 experiments ± SD and statistically analyzed using unpaired student *T*-test. For three or more groups, one-way analysis of variance was used.

## Electronic supplementary material

Supplementary Material
