# Supplementary material for: Adipocyte-activated oxidative and ER stress pathways promote tumor survival in bone via upregulation of Heme Oxygenase 1 and Survivin
Source: Sci Rep. 2018 Jan 8;8:40. doi: 10.1038/s41598-017-17800-5 (PMC5758829; doi:10.1038/s41598-017-17800-5)
Supplement: Supplementary file 1 — Supplementary Material [file 41598_2017_17800_MOESM1_ESM.pdf]

Adipocyte-activated oxidative and ER stress pathways promote tumor survival in bone via upregulation of Heme Oxygenase 1 and Survivin

Mackenzie K Herroon[1], Erandi Rajagurubandara[1], Jonathan D Diedrich[1,3], Elisabeth I Heath[2,3] and Izabela Podgorski[1,3]

[1]Department of Pharmacology and [2]Oncology; [3]Karmanos Cancer Institute, Wayne State University School of Medicine, Detroit, MI

SUPPLEMENTARY MATERIAL

SUPPLEMENTARY FIGURE LEGENDS:

Supplementary Figure 1: The cBioPortal for Cancer Genomics analysis of HO-1 (*HMOX1)* mRNA expression in patient metastatic prostate tumors from the SU2C/PCF Dream Team cohort. The plots of *HO-1* mRNA expression against the clinical attributes: "Sample Type" **(A).** and "Tumor Site" **(B).** The levels of *HO-1* expression (Y Axis) were examined across metastatic prostate cancer patients presenting in various metastatic sites, including bone (X Axis). Data are shown as log2 scaled analysis of RNA Seq Reads per Kilobase Million (RPKM).

Supplementary Figure 2: Adipocyte-induced stress response is abrogated by the treatment with 5mM NAC. (A) Western blot analysis of HO-1, BIP, and XBP1s in MDA-MB-231BO cells alone or in transwell co-culture with adipocytes, in the presence or absence of 5mM NAC. **(B-C)** Taqman RT-PCR analysis of **(B)** HO-1 and **(C)** BIP in MDA-MB-231BO cells alone or in transwell co-culture with adipocytes, in the presence or absence of 5mM NAC. Tubulin was used as a loading control for protein expression and Taqman RT-PCR analyses were normalized to HPRT1.

Supplementary Figure 3: Exposure to adipocytes does not have significant effect on the expression of SOD2 by PC3 and ARCaP(M) cells. Taqman RT PCR analysis of SOD2 expression in PC3 and ARCaP(M) cells grown alone or in transwell with adipocytes. Data were normalized to HPRT1 and are representative of triplicate experiments; n.s. = non significant changes.

Supplementary Figure 4: Adipocyte-induced stress response is abrogated by the treatment with 5mM GSH. Taqman RT-PCR analysis of **(A)** HO-1 and **(B)** BIP in PC3 cells alone or in transwell co-culture with adipocytes, in the presence or absence of 5mM glutathione (GSH). Taqman RT-PCR analyses were normalized to HPRT1.

Supplementary Figure 5. NAC or GSH treatment reduces ROS production by PCa cells. (A) ROS staining of PC3 cells in alone or transwell co-culture with adipocytes, grown in the presence or absence of 5mM NAC or 5mM GSH. **(B)** Quantified integrated fluorescence of ROS staining.

Supplementary Figure 6: Adipocyte-driven XBP1 splicing is inhibited by XBP1 inhibitors. (A) PCR analysis of spliced XBP1 **(top)** and Western blot analysis of BIP **(bottom)** in PC3 cells alone or in transwell co-culture with adipocytes, with or without 10μM Isoproterenol. **(B)** PCR analysis of spliced XBP1 in PC3 cells alone or in transwell co-culture with adipocytes, with or without 10μM or 50μM STF-083010. **(C)** PCR analysis of spliced XBP1 in PC3 cells alone or in transwell co-culture with adipocytes, with or without 5μM MKC3946. Actin was used as a loading control for semi-quantitative PCR analysis and β-Actin was used as a loading control for protein expression. For immunoblot quantitation densitometric analysis using ImageJ was performed and results are provided below each band (as fold increases relative to tumor cells Alone). Data are representative of 3 independent experiments. (**$p<0.01$, and *$p<0.05$ are considered statistically significant).

Supplementary Figure 7: PCa viability remains unchanged in the presence of NAC. (A) PC3 and **(B)** ARCaP(M) viability remains stable in increasing concentrations of NAC, up to 25mM, measured via Calcein AM assay. **(C)** Calcein staining assay of PC3 cells grown in the presence or absence of 5mM NAC or 0.4μM Staurosporine, showing no difference in cell viability at the NAC concentration used. **(D)** Western blot analysis of PC3 cells grown in the presence or absence of 5mM NAC and probed for cleaved PARP, indicating no evidence of apoptosis. Cells stressed using H_2O_2 used as positive control.

Supplementary Figure 8: HO-1 overexpression in PCa clones. (A) Protein expression of HO-1 in PC3 **(left)** and ARCaP(M) **(right)** clones with an empty vector (EV) or HO-1 (H#) inserted. Each blot shows the overexpression levels of two separate HO-1 clones. **(B)** HO-1 gene expression of PC3 **(left)** and ARCaP(M) **(right**) EV and H clones. Tubulin was used as a loading control for protein expression and Taqman RT-PCR analyses were normalized to HPRT1.

Supplementary Figure 9: Viability of PC3 cells increases with HO-1 overexpression. (A) MTT assay on PC3 EV and HO-1 clones. **(B)** PCR analysis of spliced XBP1 in PC3-EV, PC3-H3 and PC3-H9 cells; actin was used as a loading control. (**p<0.01 and *p<0.05 are considered statistically significant).

Supplementary Figure 10: Overexpression of HO-1 in PCa cells increases tumor progression and hypoxic response *in vivo*. (A) X-ray and H&E staining of mouse tibia intratibially injected with ARCaP(M)-EV or ARCaP(M)-HO1 cells and allowed to grow for 8 weeks. **(B)** Immunohistochemical analysis of BIP and CAIX in ARCaP(M)-EV- vs ARCaP(M)-HO1-bearing tibiae. Serial section without incubation with a primary antibody was used as a negative control

Supplementary Figure 11: Stress response in PCa cells upon adipocyte exposure is augmented with overexpression of HO-1. Taqman RT-PCR analysis of **(A)** CAIX, **(B)** BIP, and **(C)** XBP1 of PC3-EV and PC3-HO1 cells grown alone or in transwell co-culture with adipocytes. Taqman RT-PCR analyses were normalized to HPRT1. (*** p<0.001, **p<0.01 and *p<0.05 are considered statistically significant).

Supplementary Figure 12: Metastatic tumors in prostate cancer patients show increase in Survivin levels. Oncomine database analysis reveals upregulation of Survivin in metastatic tumors compared to primary tumors in patients in **(A)** Grasso, **(B)** Chandran, **(C)** Varambally, and **(D)** Ramaswamy databases.

Supplementary Figure 13: Full-size blots corresponding to cropped images in Figure 2A. Western blot analysis of HO-1 and BIP expression in PC3 cells alone or in transwell co-culture with bone marrow adipocytes; Tubulin was used as a loading control.

Supplementary Figure 14: Full-size blots corresponding to cropped images in Figure 2D. Western blot analysis of HO-1 and BIP in ARCaP(M) prostate cancer cells alone or in transwell co-culture with adipocytes. Actin (HO-1 blot) and Tubulin (BIP blot) were used as loading controls.

Supplementary Figure 15: Full-size blots corresponding to cropped images in Figure 4C. Western blot analysis of HO-1 and BIP in ARCaP(M) prostate cancer cells alone or in transwell co-culture with adipocytes. Actin (HO-1 blot) and Tubulin (BIP blot) were used as a loading controls. Western blot analysis of HO-1 and BIP in PC3 cells alone or in transwell co-culture with adipocytes, in the presence or absence of 5mM NAC; Tubulin was used as a loading control.

Supplementary Figure 16: Full-size blots corresponding to cropped images in Figure 8C. Western blot analysis of Bcl-xl and Survivin of PC3 cells grown alone or in transwell; β-Actin was used as a loading control.

Supplementary Figure 17: Full-size blots corresponding to cropped images in Figure 8F. Western blot analysis of Bcl-xl and Survivin of PC3-EV or PC3-HO1 cells grown either in control or transwell conditions. Tubulin was used as a loading control.

Supplementary Figure 18: Full-size blots corresponding to cropped images in Figure 9A. Western blot analysis of Survivin using PC3-EV or PC3-HO1 cells with or without 5μM ZnPP; cropped blots; β-Actin was used as a loading control.

A.

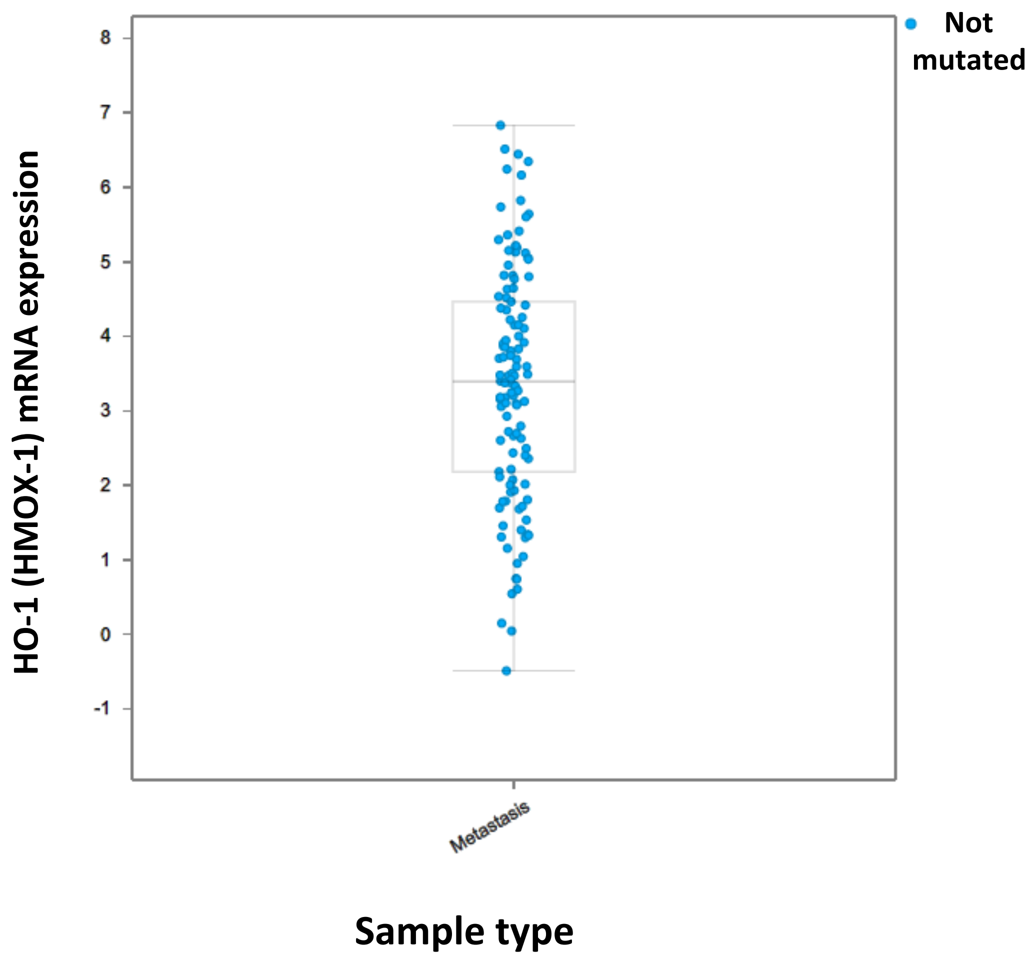

B.

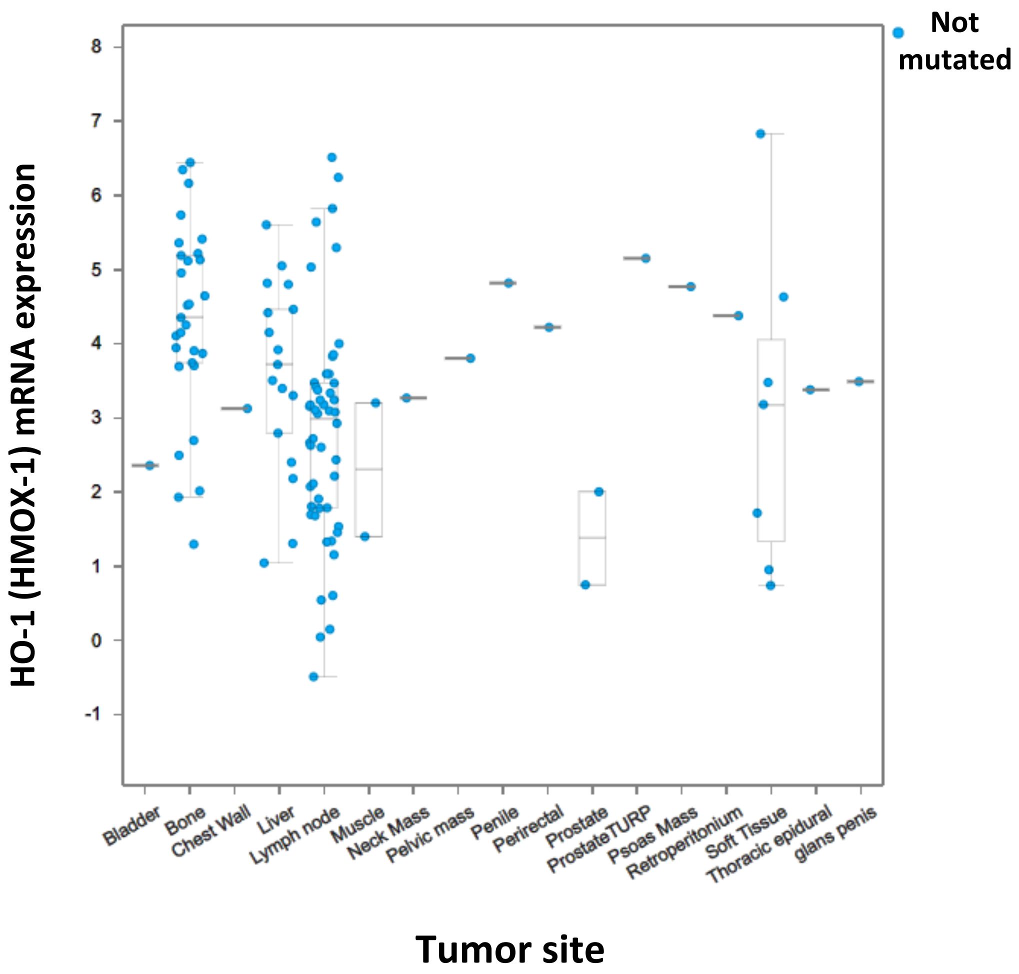

Suppl. Fig 1

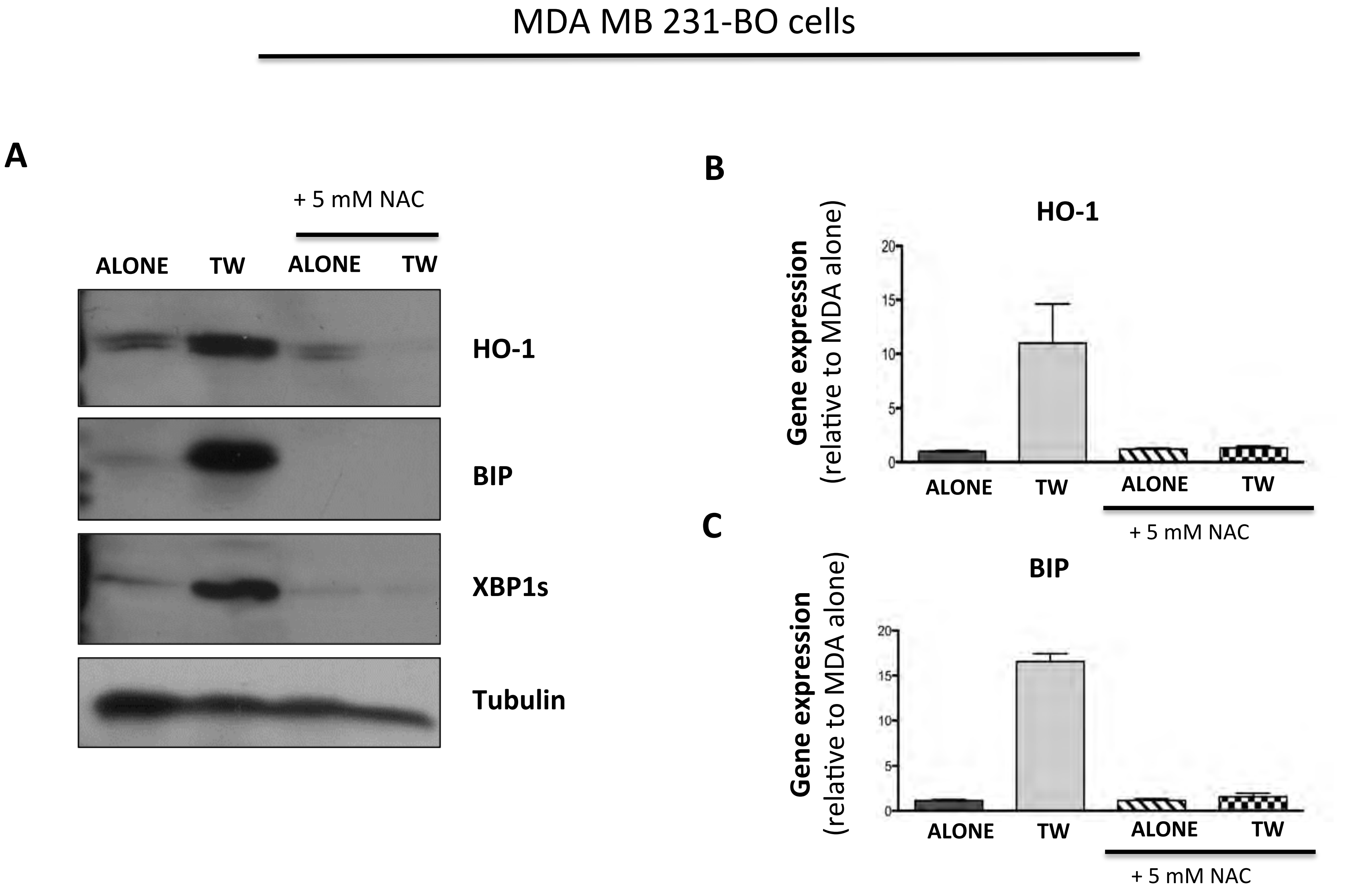
MDA MB 231-BO cells
A
+ 5 mM NAC
ALONE
TW
ALONE
TW
HO-1
BIP
XBP1s
Tubulin
B
HO-1
Gene expression
(relative to MDA alone)
20
15
10
5
0
ALONE
TW
ALONE
TW
+ 5 mM NAC
C
BIP
Gene expression
(relative to MDA alone)
20
15
10
5
0
ALONE
TW
ALONE
TW
+ 5 mM NAC

Suppl. Fig. 2

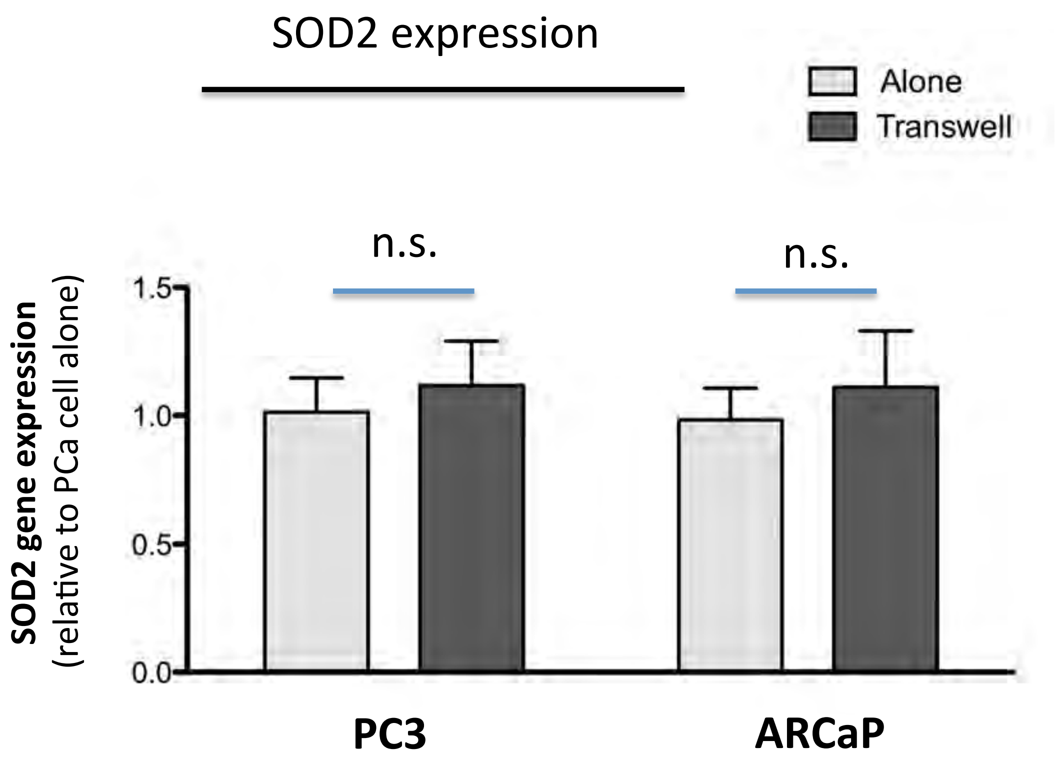

SOD2 expression
Alone
Transwell
n.s.
n.s.
SOD2 gene expression
(relative to PCa cell alone)
1.5
1.0
0.5
0.0
PC3
ARCaP

Suppl. Fig 3

A

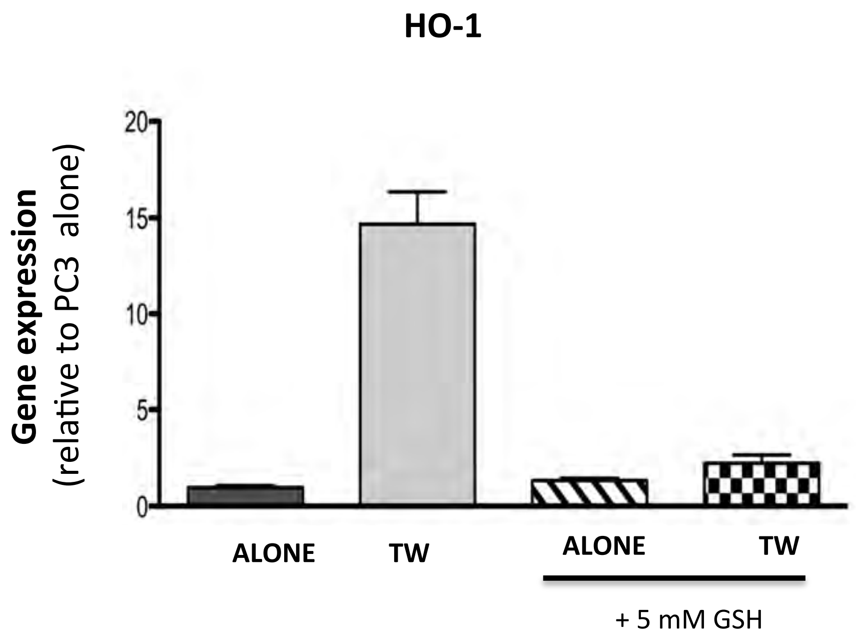

B

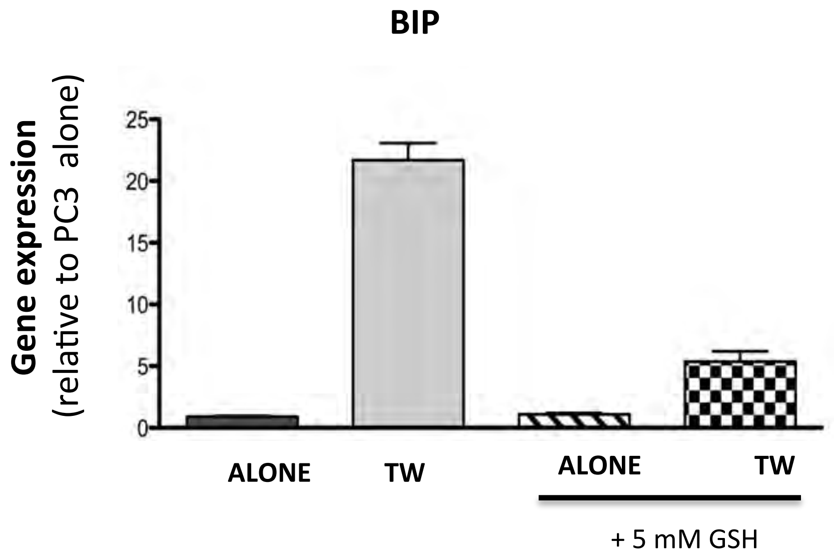

Suppl. Fig 4

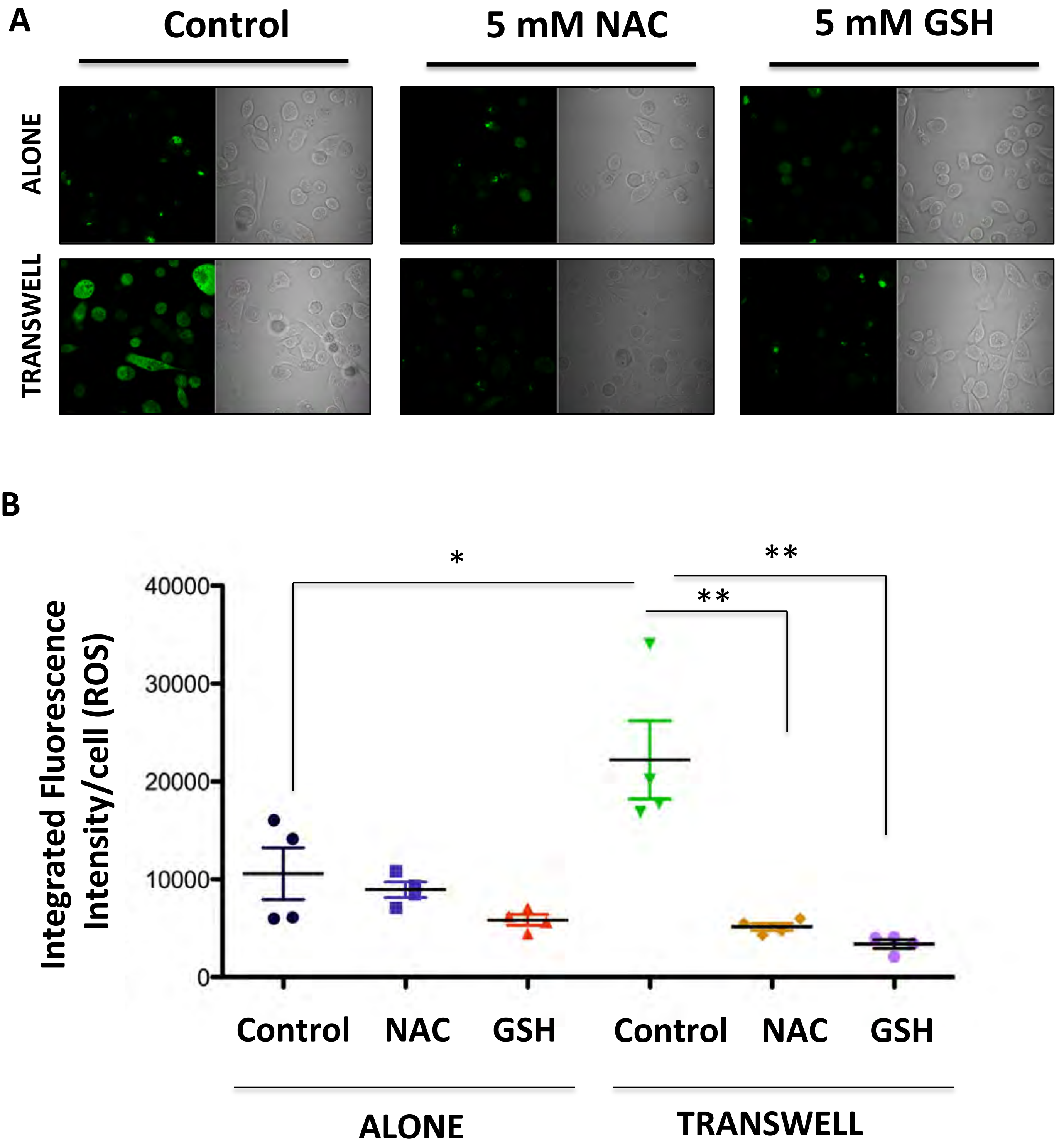
A
Control
5 mM NAC
5 mM GSH
ALONE
TRANSWELL
B
Integrated Fluorescence Intensity/cell (ROS)
40000
30000
20000
10000
0
*
**
**
Control
NAC
GSH
Control
NAC
GSH
ALONE
TRANSWELL

Suppl. Fig 5

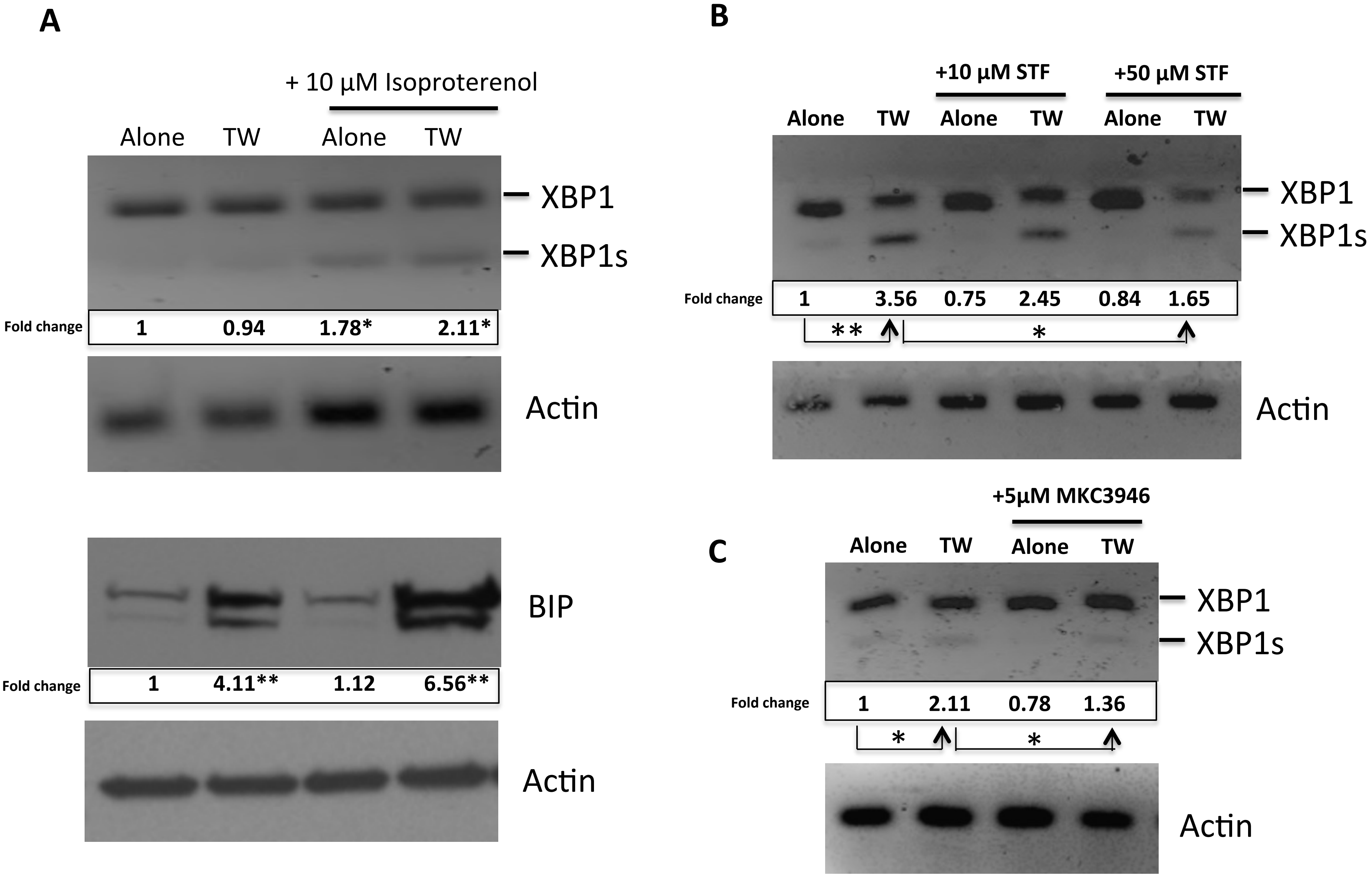
A
+ 10 μM Isoproterenol
Alone
TW
Alone
TW
XBP1
XBP1s
Fold change
1
0.94
1.78*
2.11*
Actin
BIP
Fold change
1
4.11**
1.12
6.56**
Actin
B
+10 μM STF
+50 μM STF
Alone
TW
Alone
TW
Alone
TW
XBP1
XBP1s
Fold change
1
3.56
0.75
2.45
0.84
1.65
**
*
Actin
C
+5μM MKC3946
Alone
TW
Alone
TW
XBP1
XBP1s
Fold change
1
2.11
0.78
1.36
*
*
Actin

Suppl Fig 6

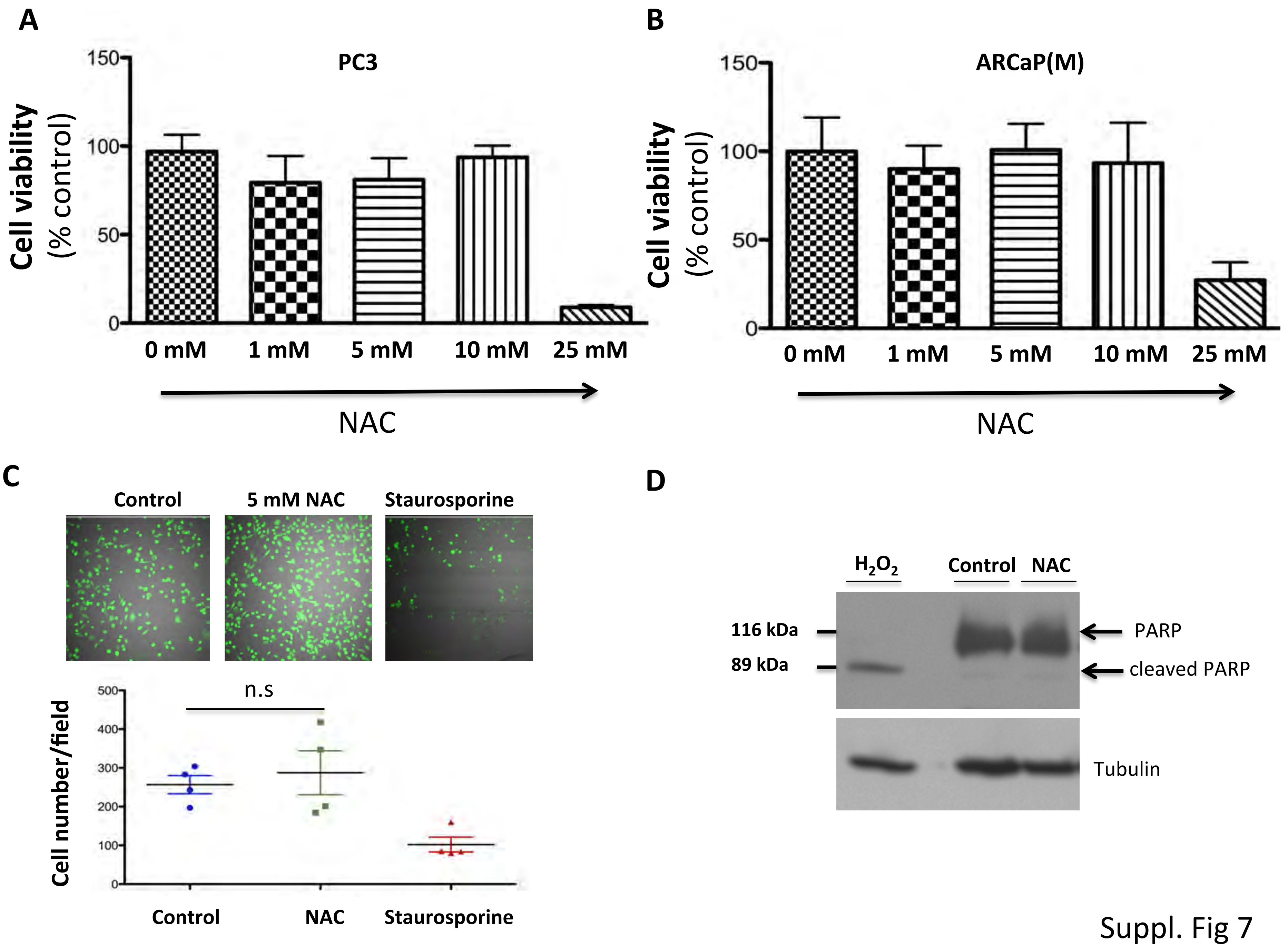
A
PC3
Cell viability (% control)
150
100
50
0
0 mM
1 mM
5 mM
10 mM
25 mM
NAC
B
ARCaP(M)
Cell viability (% control)
150
100
50
0
0 mM
1 mM
5 mM
10 mM
25 mM
NAC
C
Control
5 mM NAC
Staurosporine
n.s
Cell number/field
500
400
300
200
100
0
Control
NAC
Staurosporine
D
H2O2
Control
NAC
116 kDa
89 kDa
PARP
cleaved PARP
Tubulin

Suppl. Fig 7

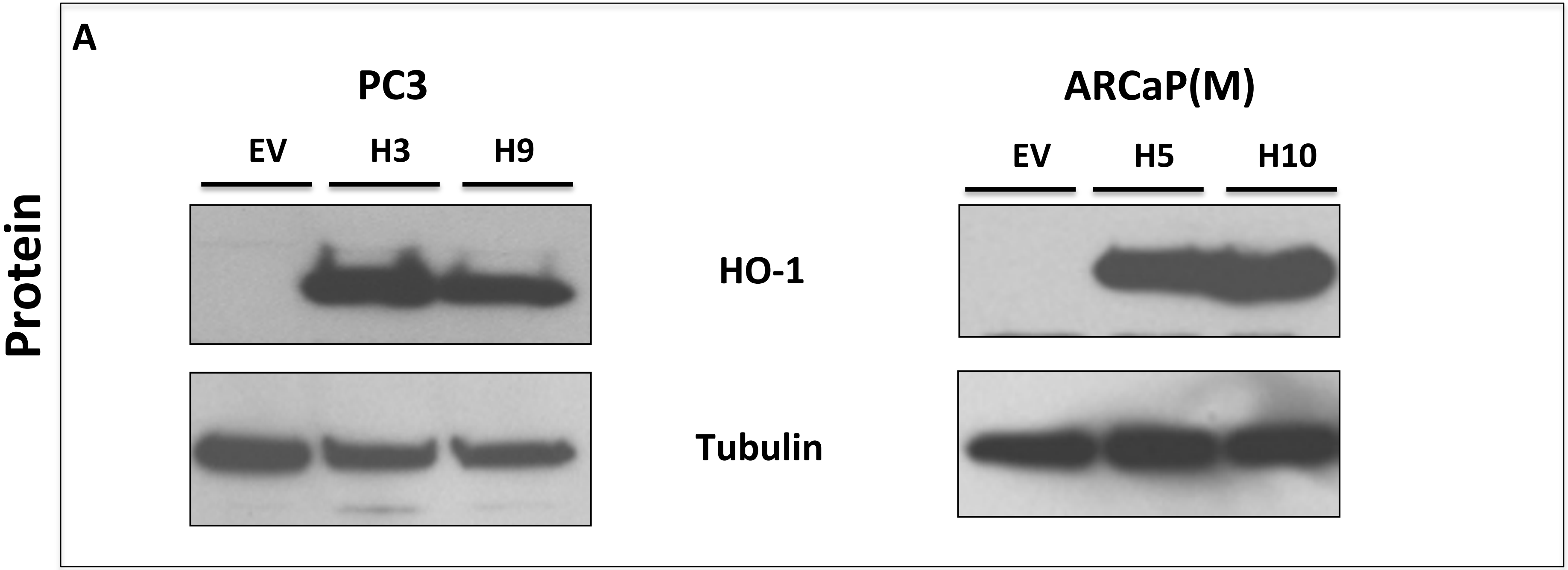
A
Protein
PC3
EV
H3
H9
ARCaP(M)
EV
H5
H10
HO-1
Tubulin

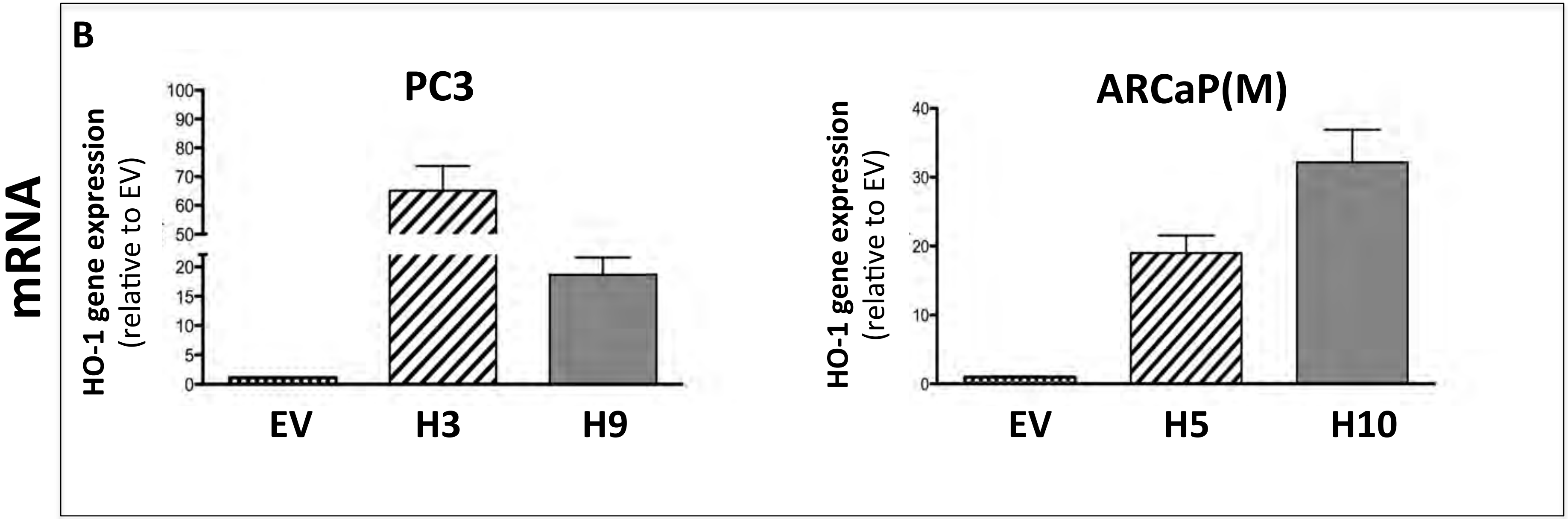
B
mRNA
PC3
HO-1 gene expression
(relative to EV)
100
90
80
70
60
50
20
15
10
5
0
EV
H3
H9
ARCaP(M)
HO-1 gene expression
(relative to EV)
40
30
20
10
0
EV
H5
H10

Suppl. Fig 8

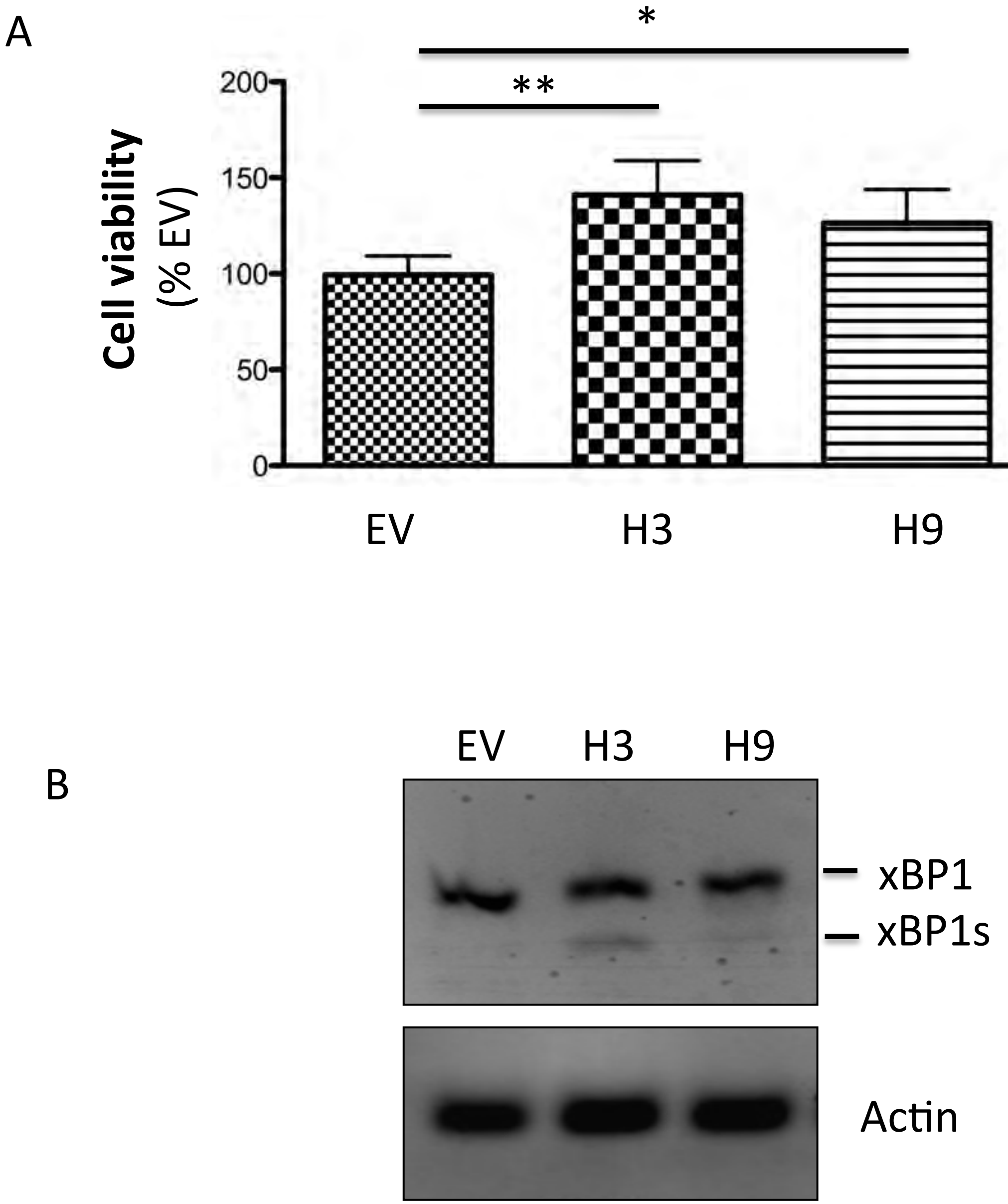

A
*
**
Cell viability
(% EV)
200
150
100
50
0
EV
H3
H9
B
EV
H3
H9
xBP1
xBP1s
Actin

Suppl. Fig 9

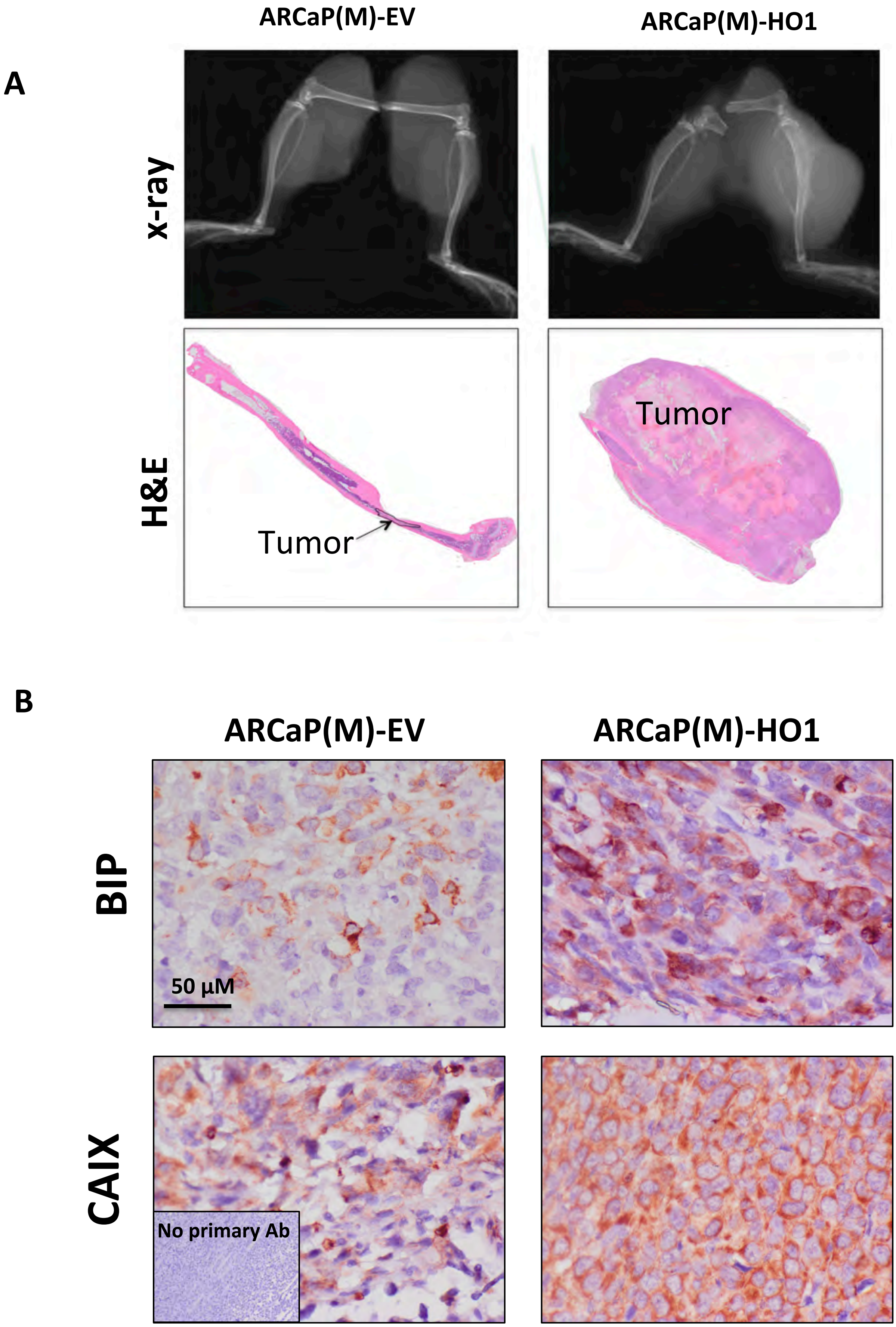
A
ARCaP(M)-EV
ARCaP(M)-HO1
x-ray
H&E
Tumor
Tumor
B
ARCaP(M)-EV
ARCaP(M)-HO1
BIP
50 µM
CAIX
No primary Ab

Suppl. Fig 10

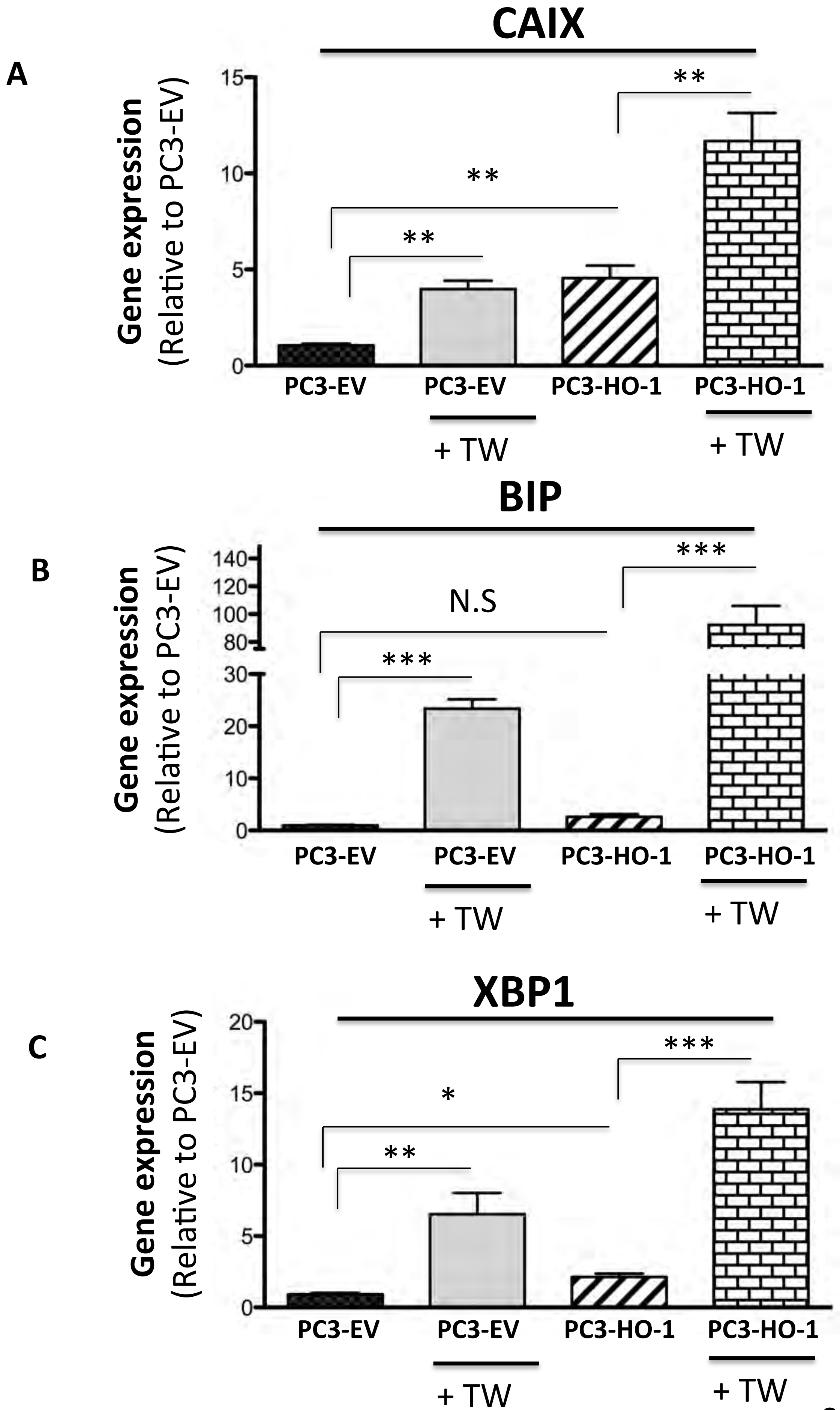
CAIX
A
Gene expression
(Relative to PC3-EV)
15
10
5
0
**
**
**
PC3-EV
PC3-EV
PC3-HO-1
PC3-HO-1
+ TW
+ TW
BIP
B
Gene expression
(Relative to PC3-EV)
140
120
100
80
30
20
10
0
***
N.S
***
PC3-EV
PC3-EV
PC3-HO-1
PC3-HO-1
+ TW
+ TW
XBP1
C
Gene expression
(Relative to PC3-EV)
20
15
10
5
0
***
*
**
PC3-EV
PC3-EV
PC3-HO-1
PC3-HO-1
+ TW
+ TW

Suppl. Fig.11

Survivin mRNA levels in primary vs. metastatic tissues from PCa patients

A

Grasso Prostate

P value: 1.07E-11
Fold increase: 6.655

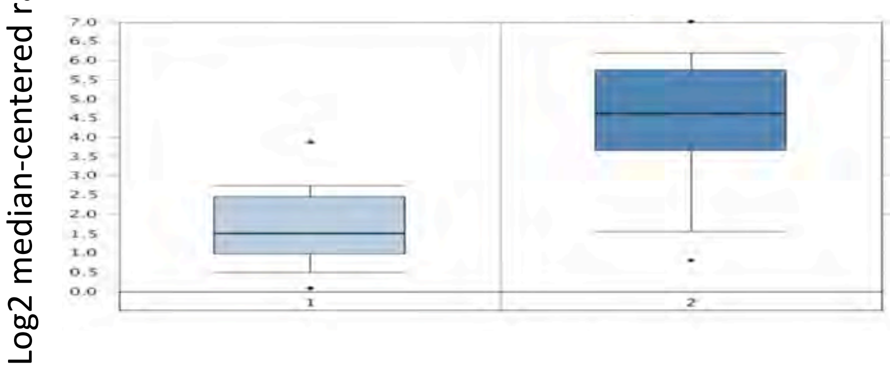

B

Chandran Prostate

P value: 8.47E-8
Fold increase: 6.516

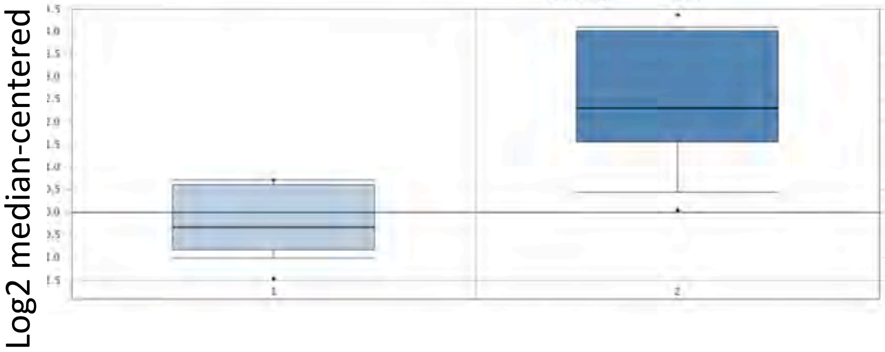

C

Varambally Prostate

P value: 1.95E-4
Fold increase: 9.508

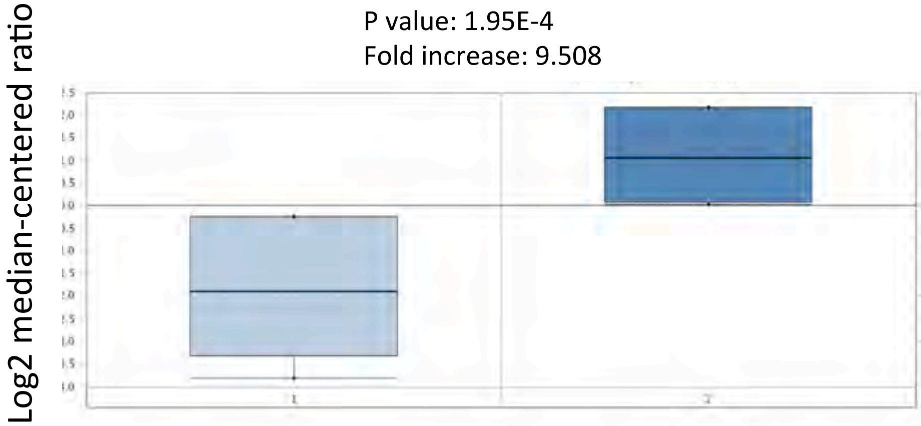

D

Ramaswamy Prostate

P value: 7.16E-4
Fold increase: 37.6378

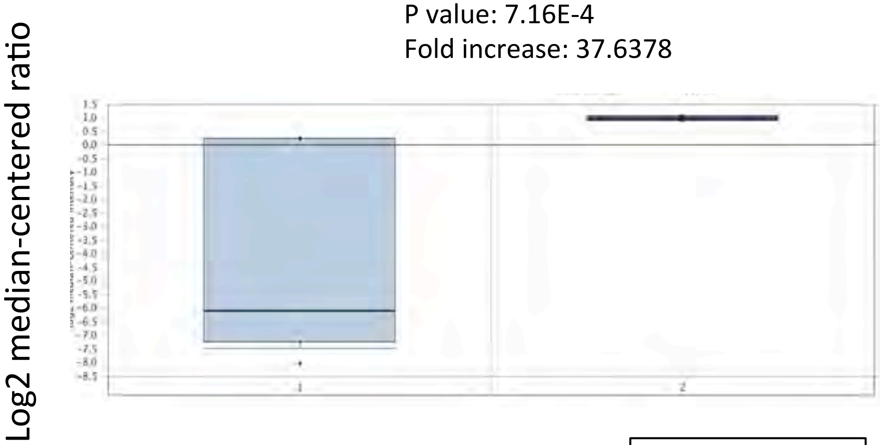

1 – primary
2-metastatic

Suppl, Fig 12

Full blots for Figure 2A

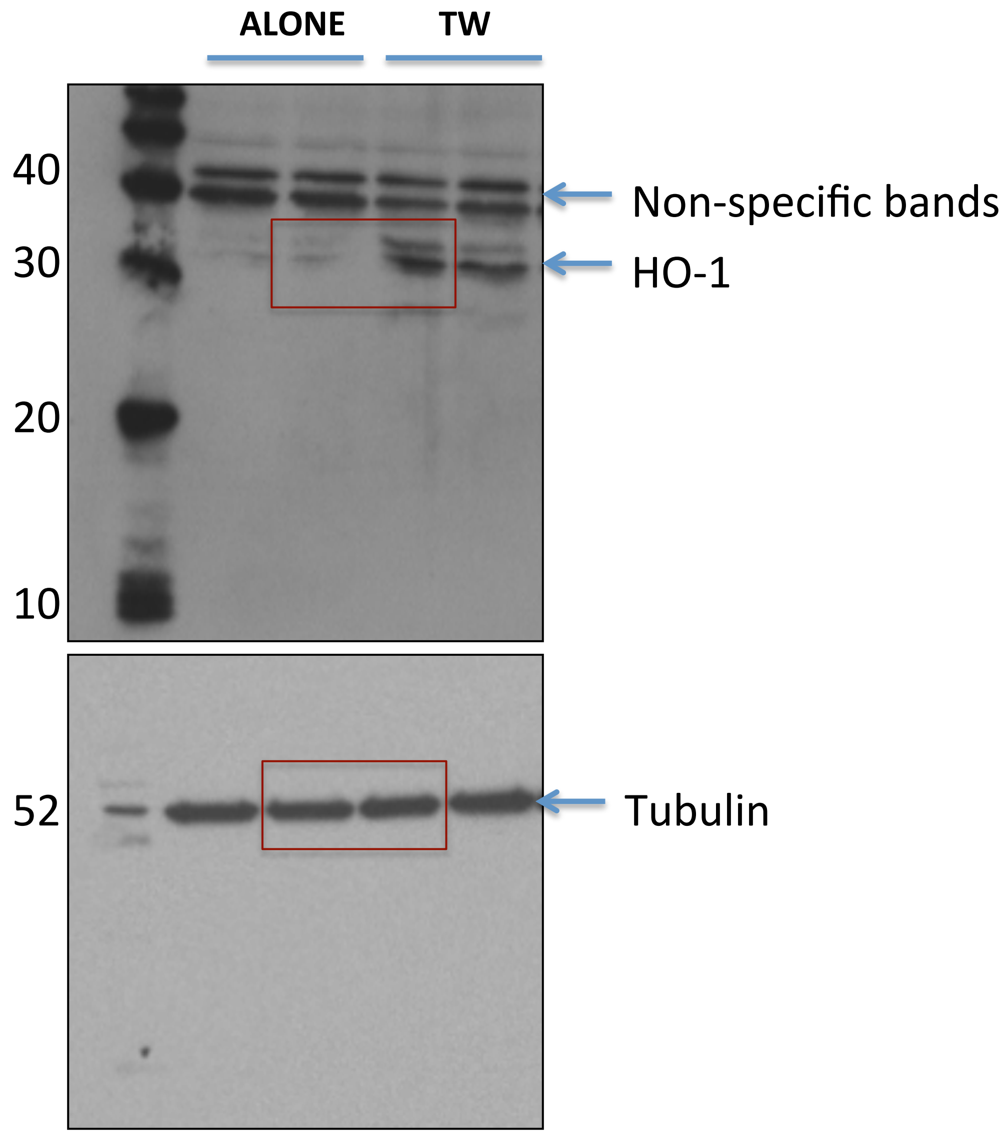

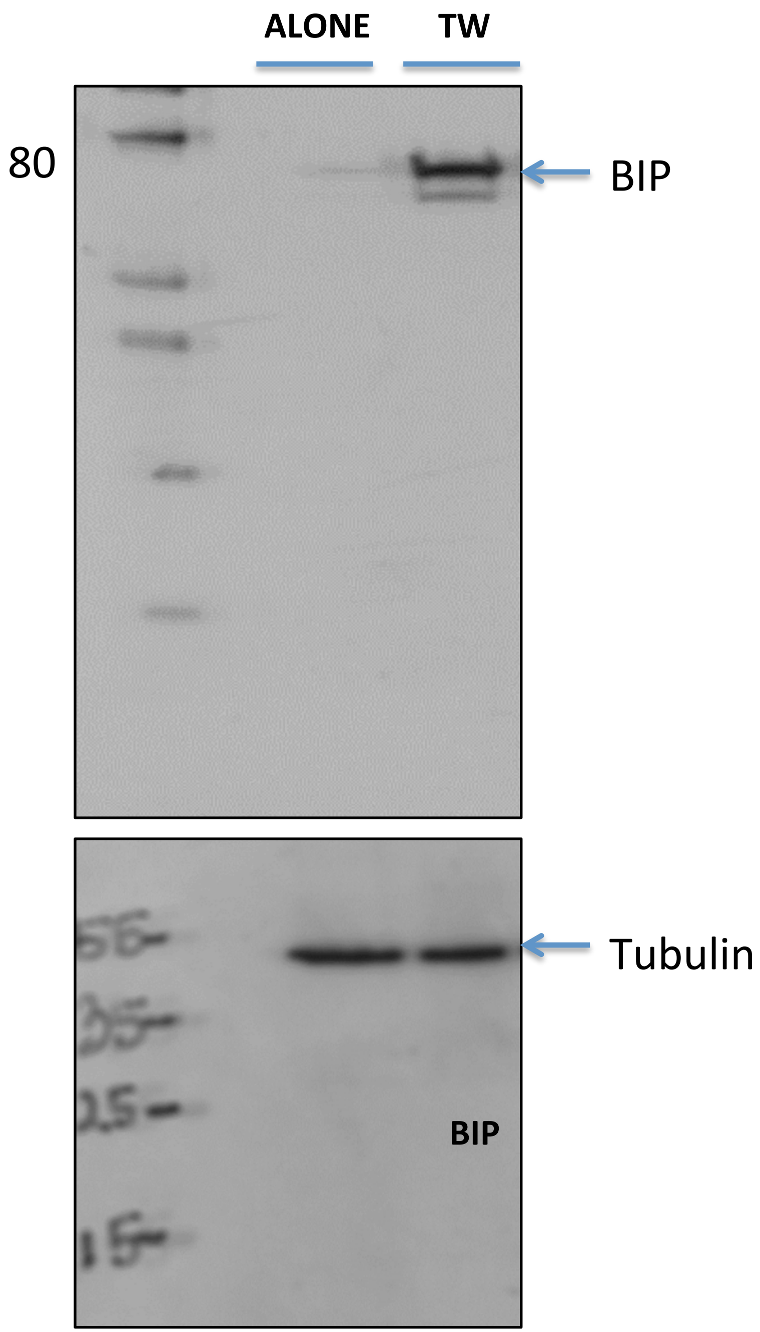

Supplementary Fig. 13

Full blots for Figure 2D

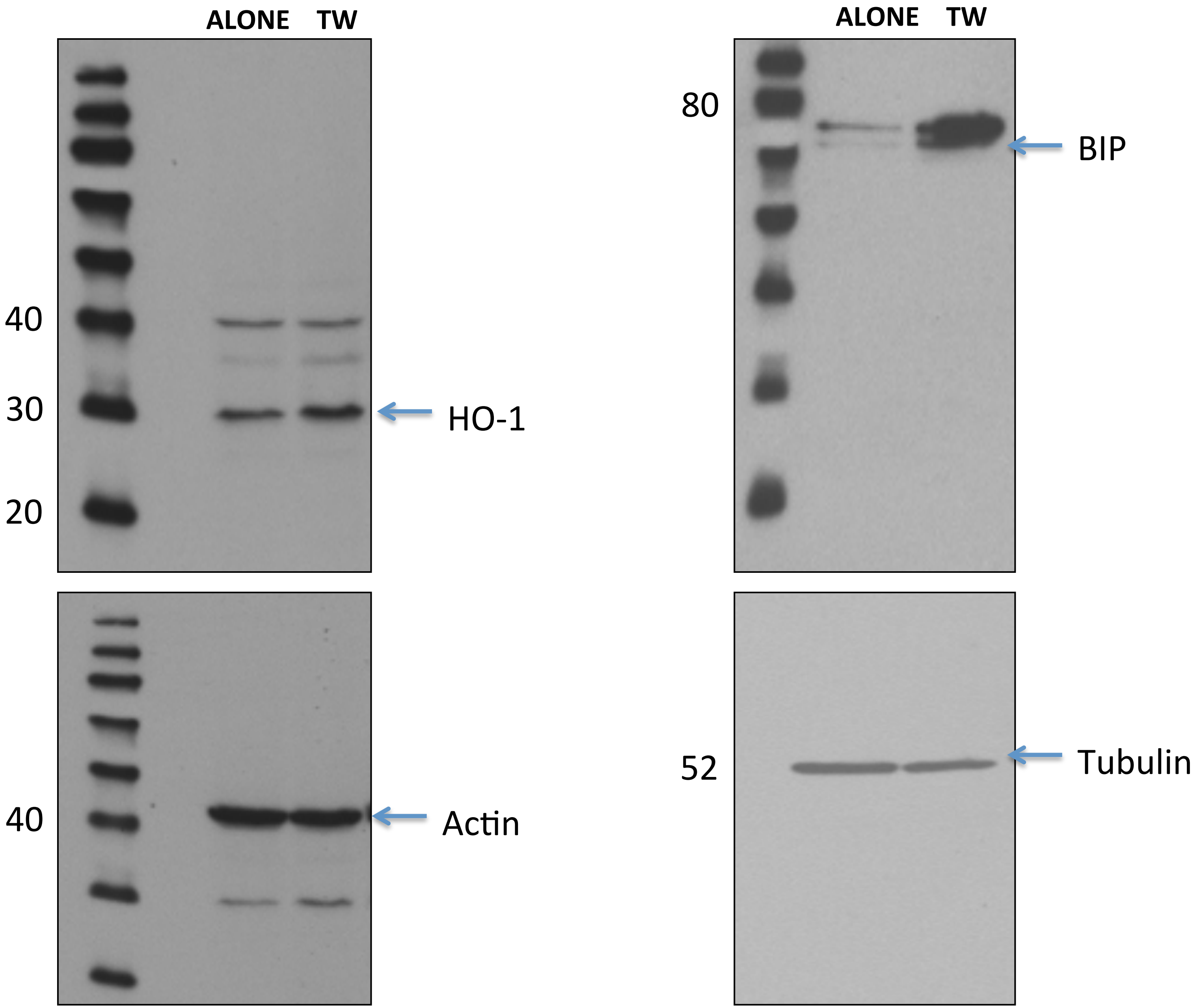

Supplementary Fig. 14

Full blots for Figure 4

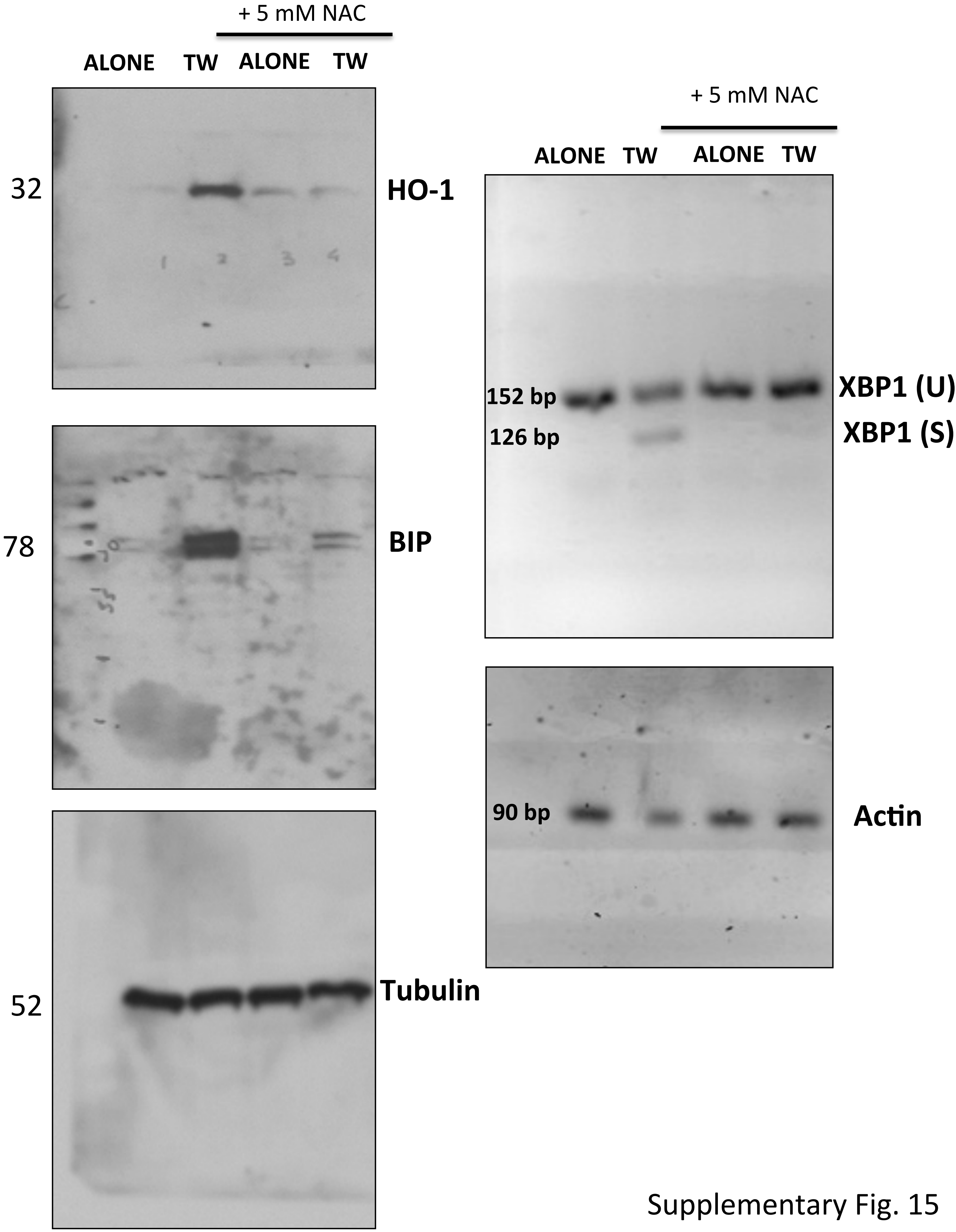

Supplementary Fig. 15

Full blots for Figure 8C

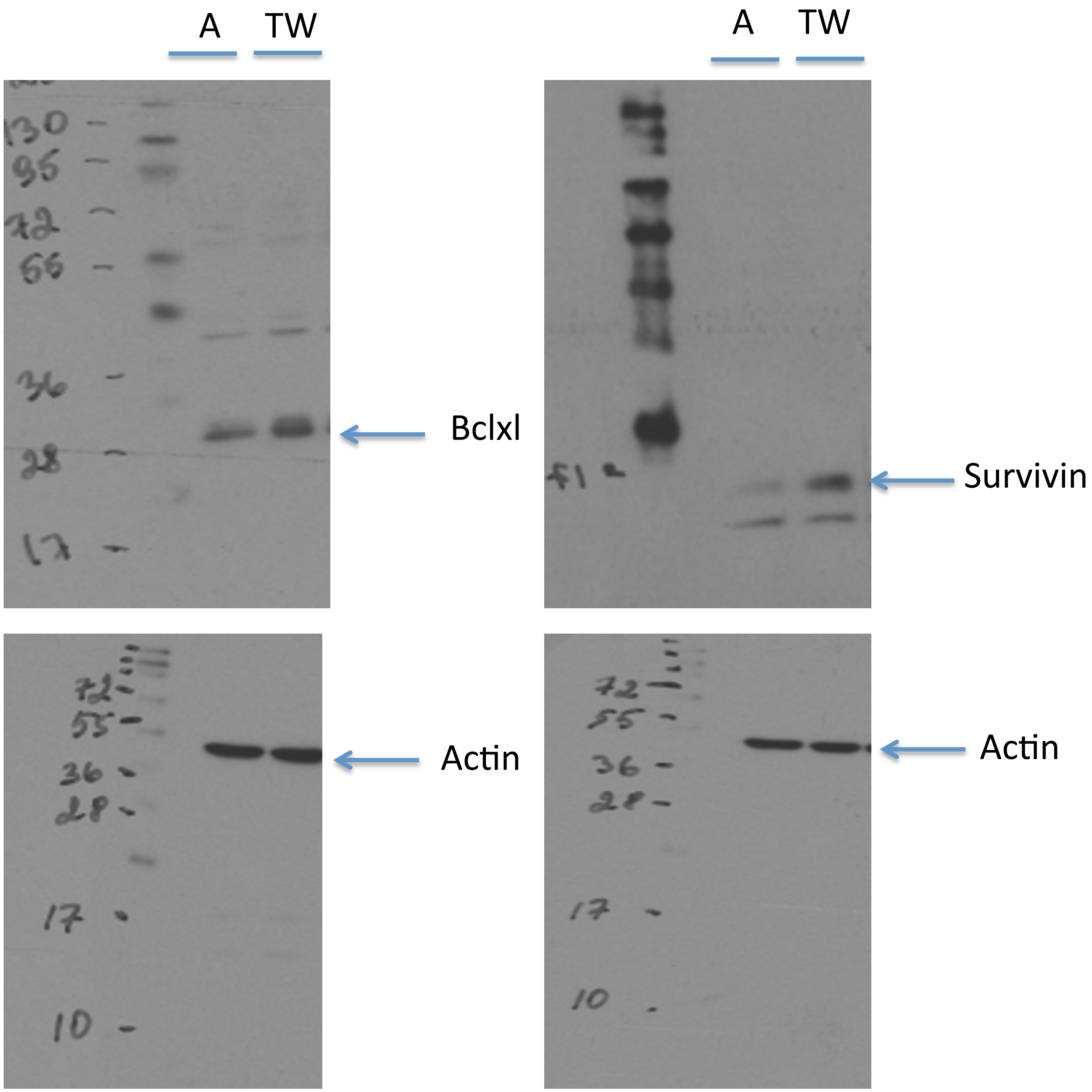

Supplementary Fig. 16

Full blots for Figure 8F

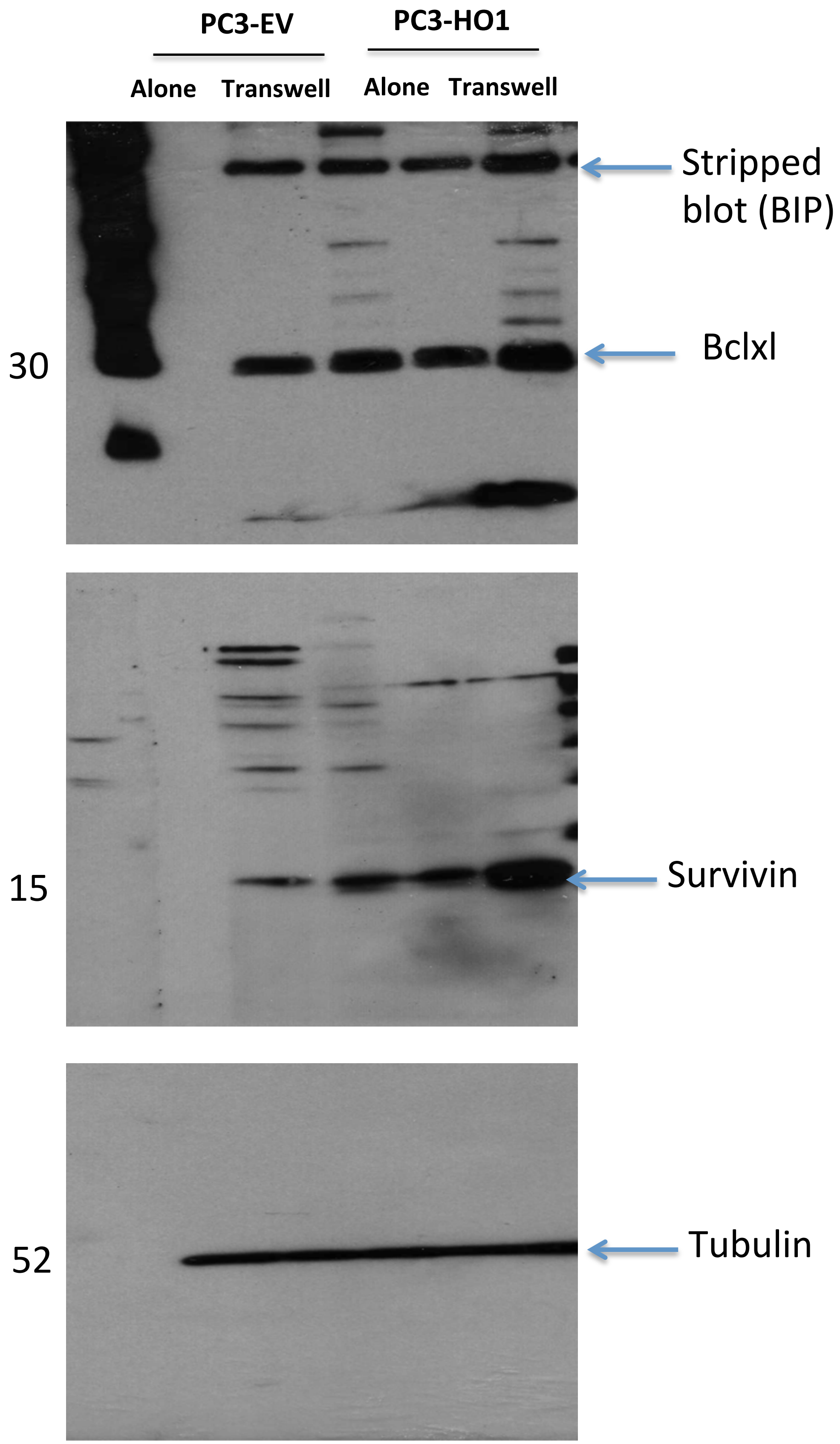

Supplementary Fig. 17

Full blots for Figure 9

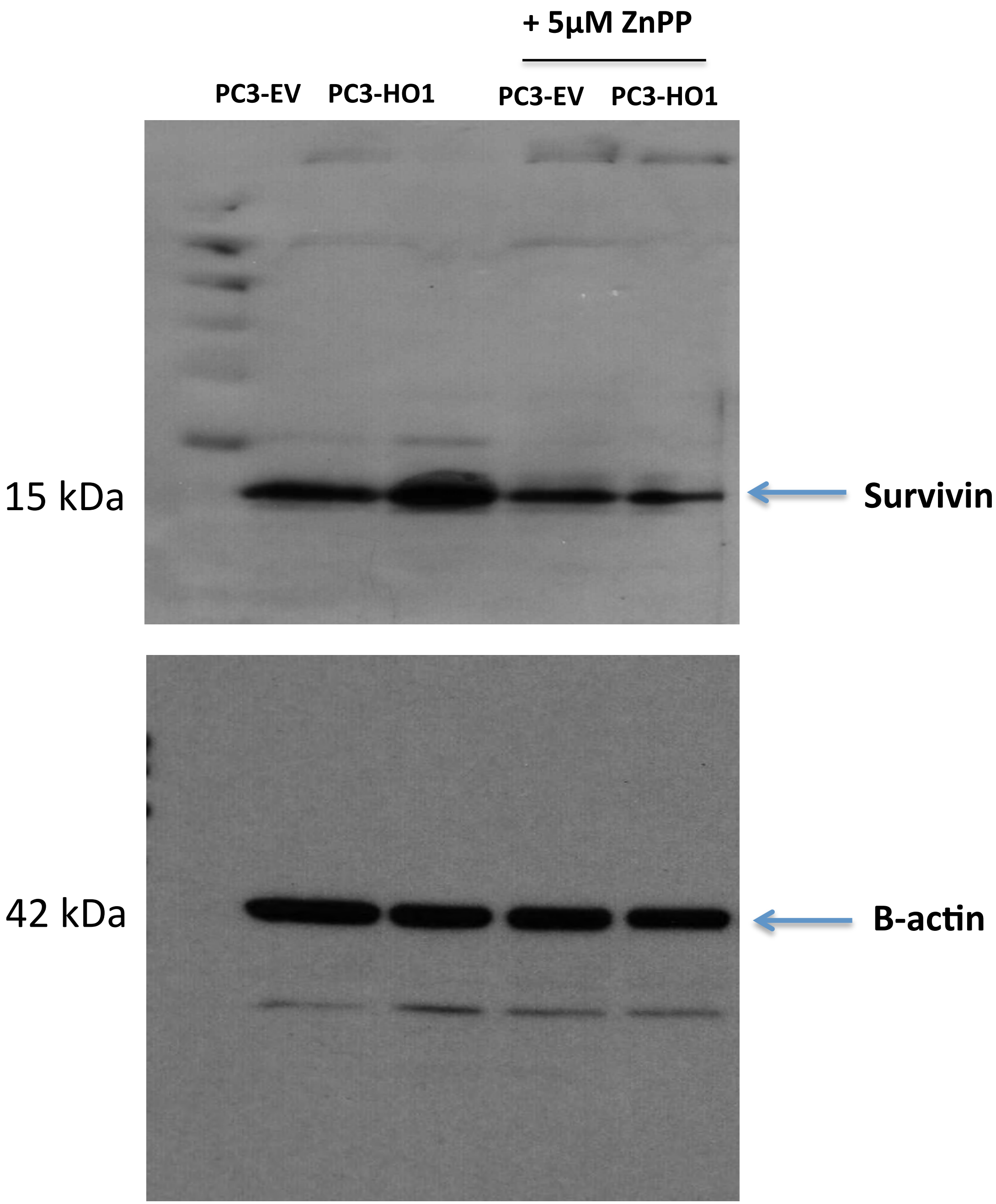

Supplementary Fig. 18
